# Health-Related Quality of Life as Assessed by the EQ-5D-5L Predicts Outcomes of Patients Treated with Azacitidine—A Prospective Cohort Study by the AGMT

**DOI:** 10.3390/cancers15051388

**Published:** 2023-02-22

**Authors:** Lisa Pleyer, Sonja Heibl, Christoph Tinchon, Sonia Vallet, Martin Schreder, Thomas Melchardt, Norbert Stute, Kim Tamara Föhrenbach Quiroz, Michael Leisch, Alexander Egle, Lukas Scagnetti, Dominik Wolf, Richard Beswick, Manuel Drost, Julian Larcher-Senn, Thomas Grochtdreis, Marc Vaisband, Jan Hasenauer, Nadja Zaborsky, Richard Greil, Reinhard Stauder

**Affiliations:** 1 Austrian Group for Medical Tumor Therapy (AGMT) Study Group, 1180 Vienna, Austria; 2 Salzburg Cancer Research Institute (SCRI), Center for Clinical Cancer and Immunology Trials (CCCIT), Austria and Cancer Cluster Salzburg (CCS), 5020 Salzburg, Austria; 3 3rd Medical Department with Hematology, Medical Oncology, Hemostaseology, Rheumatology and Infectiology, Oncologic Center, Paracelsus Medical University, 5020 Salzburg, Austria; 4 4th Medical Department of Internal Medicine, Hematology, Internistic Oncology and Palliative Medicine, Klinikum Wels-Grieskirchen GmbH, 4600 Wels, Austria; 5 Department for Hemato-Oncology, LKH Hochsteiermark, 8700 Leoben, Austria; 6 Department of Internal Medicine 2, University Hospital Krems, Karl Landsteiner Private University of Health Sciences, 3500 Krems, Austria; 7 1st Department of Internal Medicine, Center for Oncology and Hematology, Klinik Ottakring, Wiener Gesundheitsverbund, 1030 Vienna, Austria; 8 Department of Internal Medicine V, Innsbruck Medical University, 6020 Innsbruck, Austria; 9 International Marketing, Swiss Business School, 8302 Zurich, Switzerland; 10 Assign Data Management and Biostatistics GmbH, 6020 Innsbruck, Austria; 11 Department of Health Economics and Health Services Research, Hamburg Center for Health Economics, University Medical Center Hamburg-Eppendorf, 20251 Hamburg, Germany; 12 Life and Medical Sciences Institute, University of Bonn, 53115 Bonn, Germany; 13 Laboratory of Immunological and Molecular Cancer Research (LIMCR), 5020 Salzburg, Austria

**Keywords:** health-related quality of life (HRQoL), patient-reported outcome (PRO), EuroQol 5-Dimension 5-Level questionnaire (EQ-5D-5L), azacitidine, Austrian Registry of Hypomethylating Agents, prognosis, mixed-effects linear models, acute myeloid leukaemia, myelodysplastic syndromes, chronic myelomonocytic leukaemia

## Abstract

**Simple Summary:**

The EuroQol 5-Dimension 5-level (EQ-5D-5L) questionnaire is a globally used and multiply validated tool to assess health-related quality of life (HRQoL), but data on its use for patients with myeloid neoplasias is scarce. The aim of this prospective cohort study was to alleviate this knowledge gap. Our data show in a homogenous population of azacitidine-treated patients for the first time that (1) myeloid patients have significantly worse HRQoL than a population norm (i.e., a representative sample of the German general adult population) from a similar geographic region, matched by age, sex and number of comorbidities; (2) The EQ-5D-5L questionnaire response provides added prognostic value to the International Prognostic Scoring System (IPSS) and the revised IPSS (R-IPSS), which are longstanding gold standards of prognostication in these diseases; (3) the multivariate-adjusted significant predictive value of the EQ-5D-5L response parameters on patient outcomes including response to azacitidine, time to next treatment and overall survival; (4) longitudinal assessment of the EQ-5D-5L response/clinical parameter pairs revealed significant additional, independent associations.

**Abstract:**

In this prospective study (NCT01595295), 272 patients treated with azacitidine completed 1456 EuroQol 5-Dimension (EQ-5D) questionnaires. Linear mixed-effect modelling was used to incorporate longitudinal data. When compared with a matched reference population, myeloid patients reported more pronounced restrictions in usual activities (+28%, *p* < 0.0001), anxiety/depression (+21%, *p* < 0.0001), selfcare (+18%, *p* < 0.0001) and mobility (+15%, *p* < 0.0001), as well as lower mean EQ-5D-5L indices (0.81 vs. 0.88, *p* < 0.0001), and lower self-rated health on the EuroQol Visual Analogue Scale (EQ-VAS) (64 vs. 72%, *p* < 0.0001). After multivariate-adjustment, (i) the EQ-5D-5L index assessed at azacitidine start the predicted time with clinical benefit (TCB) (9.6 vs. 6.6 months; *p* = 0.0258; HR = 1.43), time to next treatment (TTNT) (12.8 vs. 9.8 months; *p* = 0.0332; HR = 1.42) and overall survival (OS) (17.9 vs. 12.9 months; *p* = 0.0143; HR = 1.52); (ii) Level Sum Score (LSS) predicted azacitidine response (*p* = 0.0160; OR = 0.451) and the EQ-5D-5L index showed a trend (*p* = 0.0627; OR = 0.522); (iii) up to 1432 longitudinally assessed EQ-5D-5L response/clinical parameter pairs revealed significant associations of EQ-5D-5L response parameters with haemoglobin level, transfusion dependence and hematologic improvement. Significant increases of the likelihood ratios were observed after addition of LSS, EQ-VAS or EQ-5D-5L-index to the International Prognostic Scoring System (IPSS) or the revised IPSS (R-IPSS), indicating that they provide added value to these scores.

## 1. Introduction

Azacitidine is the first treatment to be associated with improved overall survival (OS), and to be approved by both US and European regulatory authorities for the treatment of patient subgroups with myeloid neoplasms. In patients with myelodysplastic syndromes (MDS) [1,2] and chronic myelomonocytic leukaemia (CMML) [3], it remains the only approved disease-modifying therapeutic substance, whereas several new drugs have recently been approved for certain patient subgroups with acute myeloid leukaemia (AML).

Globally, there has been a distinctive shift towards taking patient perspectives into account when making (regulatory) healthcare and treatment decisions. Traditional clinical ways of measuring health and the effects of treatment are thus increasingly being accompanied by patient-reported outcome measures. In the broad field of the latter, the generic EuroQol 5-Dimension (EQ-5D) questionnaire is multiply validated and globally has been the most used tool in many areas of medicine, including oncology, for over three decades.

Although regulatory agencies offered guidance for the use of patient-reported outcome measures to support labelling claims as early as 2005 [4,5,6,7], the European LeukemiaNet pointed to the importance of assessing HRQoL in the clinical management of patients with MDS in 2013 [8], and the EQ-5D has been the preferred measure of HRQoL for the UK National Institute for Health and Care Excellence since 2008, published reports on HRQoL data in MDS, CMML and AML are scarce. There are 4 publications in MDS, and 10 in AML, 13 of which only report on the mean EQ-VAS and/or the mean or median EQ-5D-5L index value. Only one report assessed the impact of the EQ-5D-5L index on a time-to-event endpoint [9], and only one publication provided details on non-composite results [10], both in patients with lower-risk MDS (Table 1). Publications correlating EQ-5D-5L measures with treatment outcomes in general, and with azacitidine-related outcomes in particular, are lacking to date. The only detailed EQ-5D-5L data on this topic stem from this report.

In this prospective study, we compared EQ-5D-5L responses between patients with MDS, CMML and AML and a population norm (i.e., a representative sample of the German general adult population without myeloid (or other) neoplasias) from a similar geographic region, matched by age, sex and number of comorbidities. In myeloid patients treated with azacytidine within the Austrian Registry of Hypomethylating Agents, we performed more detailed analyses and assessed (1) whether EQ-5D-5L composite variables provided added value to the (R)-IPSS; (2) there might be a predictive value of EQ-5D-5L composite variables (including LSS, EQ-VAS and EQ-5D-5L index value) on the response to azacitidine and several time-to-event endpoints; and (3) performed longitudinal assessments of EQ-5D-5L response/clinical parameter pairs.

## 2. Materials and Methods

### 2.1. Study Design and Participants

**Figure 1:**
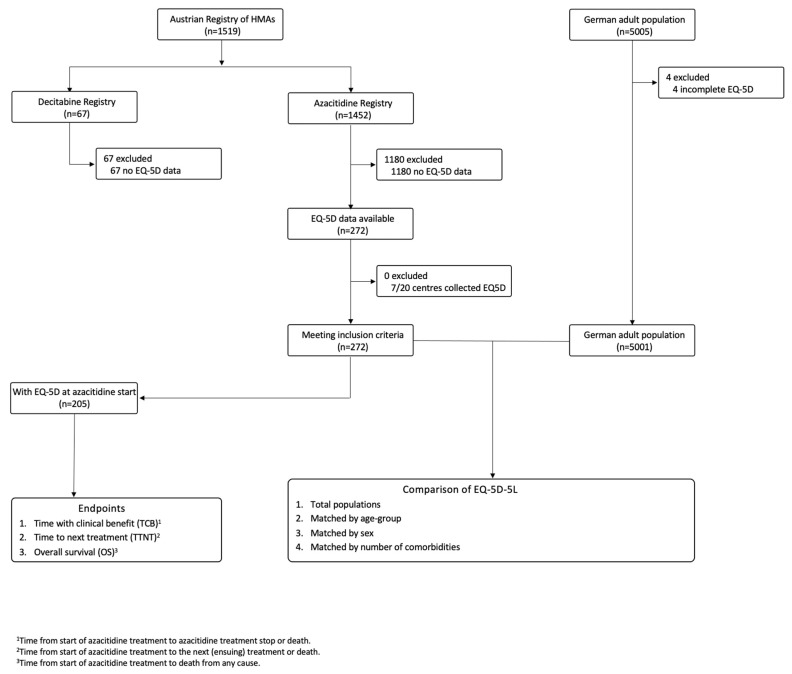
**Consort diagram.** The data collection and cleaning period was from 2 February 2012 to 3 March 2022. Database lock (last patient in) was on 13 December 2020. The inclusion criteria were (1) the diagnosis of MDS, CMML or AML, which was independently and centrally verified on the basis of submitted data; (2) treatment with azacitidine; (3) inclusion in the Austrian Registry of Hypomethylating agents; (4) the presence of a written informed consent for all patients alive at the time of data entry; (5) age ≥ 18 years; (6) the completion of at least one EQ-5D questionnaire. No data from patients <18 years were received. No patients fulfilling these criteria were excluded from the analyses. A total of 6 of 1456 (0.4%) of EQ-5D questionnaires were excluded (empty questionnaire). Permissions to use the German version of EQ-5D questionnaires was obtained from EuroQol. All data for this study were collected prospectively. This study has been reported according to the Strengthening the Reporting of Observational Studies in Epidemiology (STROBE) statement. To ensure uniformity, composite variables based on provided data were allocated for each individual patient at the start of azacitidine treatment, including diagnosis of MDS, CMML or AML according to the WHO 2016 diagnostic criteria [25], cytogenetic risk group according to the International Prognostic Scoring System (IPSS) [26] and the revised IPSS (R-IPSS) [27] and the IPSS and R-IPSS risk categories themselves.

In this prospective cohort study, data from non-selected, consecutive patients were provided by seven Austrian centres (Supplementary p. 1) participating in the Austrian Registry of Hypomethylating Agents of the Austrian Group for Medical Tumour Therapy (AGMT) Study Group (NCT01595295; ethics committee approval 415-EP/39/Feb-2009; details published previously [23,24]; Figure 1).

The EQ-5D consists of five questions (also known as dimensions (5D): mobility, selfcare, usual activities, pain/discomfort, anxiety/depression) with 5 levels (5L) of problem severity in the responses, as well as a Visual Analogue Scale (EQ-VAS) aiming to capture a respondents’ rating of their ‘health today’ on a scale from 0–100. The composite scores Level Sum Score (LSS) and EQ-5D-5L index are explained in Supplementary p. 2. The EQ-5D questionnaires were assessed at the start of azacitidine treatment cycles. The EQ-5D-5L results of patients diagnosed with MDS, CMML or AML were compared with those of a German population norm (i.e., a representative sample of the German general adult population without myeloid (or other) neoplasias) [28]. The EQ-5D-5L German value set [29] and the reverse crosswalk tool provided by EuroQol on November 16, 2020 were used for calculation of the EQ-5D-5L indices (Supplementary p. 3).

Patients with an EQ-5D available at azacitidine treatment start were stratified according to their LSS, EQ-VAS or EQ-5D-5L index being </≥ the respective group median.

### 2.2. Statistical Analyses

Only observed values were analysed. Baseline and treatment-related factors were compared using the χ^2^ test for categorical variables and the Wilcoxon test for continuous variables. Patient subgroups were compared using the log-rank test. All *p*-values and 95% CIs are two-sided. The threshold for statistical significance was 0.05. Time-to-event endpoints were analysed using the Kaplan–Meier method.

Proceeding in analogy to Efficace et al. [30] who demonstrated that self-reported fatigue provided added value to the IPSS and R-IPSS in patients with MDS, the likelihood ratio (LHR) test was used to determine whether EQ-5D-5L response parameters provided added value to the IPSS or R-IPSS.

The prognostic information provided by LSS, EQ-VAS and EQ-5D-5L index, with regards to whether a patient is likely to respond to azacitidine or not was assessed by univariate and multivariate-adjusted logistic regression analyses. Cox regression models for time-to-event endpoints were applied.

To identify variables that might be associated with patient-reported outcomes, linear mixed-effect modelling was utilised, with patient identity as the grouping variable. *p*-values were visualised using heatmaps.

Sensitivity analyses were performed to check the general conclusions by assessing different endpoints (response subtypes, OS, TCB, TTNT), and assessing both continuous and dichotomised variables. The definition of outcomes and further statistical details are given in Supplementary pp. 4–7.

Assign Data Management and Biostatistics GmbH performed statistical analyses with SAS^®^ 9.4. The Life & Medical Sciences Institute, University of Bonn performed statistical analyses including mixed-effect linear modelling with Python 3.8.12.

## 3. Results

### 3.1. Myeloid Patient Characteristics

Data from 272 patients diagnosed with MDS, CMML or AML who were treated with azacitidine between 21 May 2007 and 21 December 2020 were prospectively analysed (Figure 1). Of these, 205 had filled out an EQ-5D at azacitidine treatment start (Figure 1). This subset was used for time-to-event endpoint analyses.

Myeloid patient characteristics at azacitidine treatment start by EQ-5D group are shown in Supplementary p. 8. In the group, 129 (47%), 33 (12%) and 110 (40%) of 272 patients had MDS, CMML or AML, respectively. A total of 168 (62%) of 272 patients were male, the median age was 74.0 (IQR 69.0–79.0) years, 33 (12%) had treatment-related disease, 51 (19%) had an ECOG performance score of ≥2 and median bone marrow blasts were 12% (IQR 5–35%). Differential blood count and other lab values of the EQ-5D group are shown in Supplementary pp. 9–10. A further 86 (32%) and 35 (13%) of 272 patients were red blood cell and/or platelet transfusion dependent, respectively (Supplementary pp. 9–10). Finally, 205 (75%) of 272 patients had at least one additional comorbidity (Supplementary p. 11).

Azacitidine treatment and response characteristics are shown in Supplementary pp. 12–13. Median follow-up duration from diagnosis was 23.4 months (IQR 12.3–40.9) and from azacitidine treatment start 14.7 months (7.8–26.7).

### 3.2. Patients Treated with Azacitidine Reveal Profound Impairments in HRQoL

Supplementary pp. 14–15 show the most frequent response patterns for questionnaires filed out at azacitidine treatment start and for all EQ-5D questionnaires. Supplementary p. 16 gives an overview of the EQ-5D responses by patient group, response status and number of azacitidine treatment cycles. The mean number of filled-out EQ-5D questionnaires per patient was 5.4 (SD 6.2), the median number was 3.0 (IQR 1.0–3.0).

The myeloid cohort (*n* = 272) was characterised by mean (SD) LSS, EQ-5D-5L index value and EQ-VAS of 9.1 (3.9), 0.807 (0.232) and 63.9 (21.7), respectively, in their first available EQ-5D-5L questionnaire; results were similar when focusing on patients who had filled out an EQ-5D at azacitidine treatment start (*n* = 205) (Table 2). In this subgroup, problems (slight, moderate, severe or extreme) were self-reported in the dimensions of mobility (104 (51%) of 205), selfcare (46 (22%)), usual activities (120 (59%)), pain/discomfort (102 (50%)) and anxiety/depression (100 (49%)).

**Figure 2:**
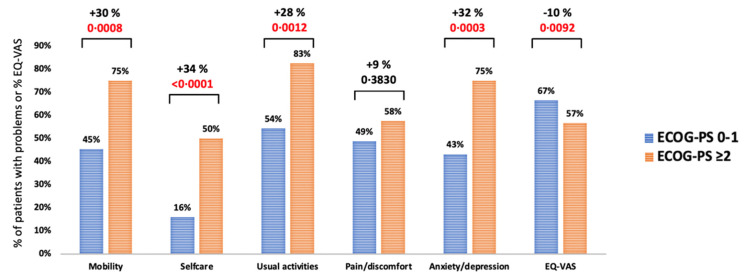
EQ-5D-5L responses available at azacitidine treatment start (*n* = 205), stratified by ECOG-PS.

The following parameters at azacitidine treatment start significantly correlated with adverse EQ-5D-5L responses: monocytes ≥10%, haemoglobin levels <10 g/dL, >3 red blood cell transfusions prior to azacitidine start, Eastern Cooperative Oncology Group Performance Status (ECOG-PS) of ≥2, high risk Hematopoietic Cell Transplantation-specific Comorbidity Index (HCT-CI) (Table 2). For example, patients with an ECOG-PS ≥2 experienced more significantly problems in the dimensions of mobility (+30%, *p* = 0.0008), selfcare (+34%, *p* < 0.0001), usual activities (+28%, *p* = 0.0012) and anxiety/depression (+32%, *p* = 0.0003), and had significantly reduced EQ-VAS (−10%, *p* = 0.0092) (Figure 2).

### 3.3. Comparison of HRQoL with a Reference Population Matched by Age, Sex and Number of Comorbidities

We compared HRQoL of the myeloid cohort with that of a German population norm (i.e., a representative sample of the German general adult population without myeloid (or other) neoplasias) [28] with a similar ethnical and socioeconomic background (Figure 1). Myeloid patients reported more pronounced restrictions in mobility (51 vs. 35%, *p* < 0.0001), selfcare (25 vs. 7%, *p* < 0.0001), usual activities (56 vs. 28%, *p* < 0.0001) and anxiety/depression (+15%, *p* < 0.0001), as well as lower mean EQ-5D-5L indices (0.81 vs. 0.88, *p* < 0.0001) and lower self-rated health on EQ-VAS (64 vs. 72%, *p* < 0.0001) than the German population norm (Table 3). These significant differences could also be observed after stratification by age group, sex or number of comorbidities (Table 3).

### 3.4. IPSS and R-IPSS Prognosticate OS and TTNT

Myeloid patients with lower-risk IPSS had significantly longer unadjusted survival than patients with higher-risk IPSS (21.0 months [95% CI 14.6–30.3] vs. 12.8 months [10.2–16.9]; HR = 0.62 [0.44–0.88]; LHR 7.32; *p* = 0.0068). Similarly, patients with lower-risk R-IPSS had significantly longer unadjusted survival than patients with higher-risk R-IPSS (30.3 months [11.2–39.3] vs. 14.6 months [11.9–17.8]; HR = 0.561 [0.3320.949]; LHR 5.37; *p* = 0.0205) (Table 4, first four columns).

Patients with lower-risk IPSS showed a trend towards longer TTNT (*p* = 0.0578), and patients with lower-risk R-IPSS showed significantly longer TTNT than their higher-risk counterparts (17.6 months [6.9–37.7] vs. 10.8 months [9.3–12.6]; HR = 0.615 [0.379–1.000]; LHR 4.31; *p* = 0.0379) (Table 4, first 4 columns).

### 3.5. EQ-5D-5L Composite Scores at Azacitidine Start Provide Added Value to the (R)-IPSS

For the endpoint OS, significant increases of the likelihood ratio (LHR) were observed after addition of (i) the LSS to the IPSS (LHR increased from 7.32 to 10.69; *p* = 0.0048) or the R-IPSS (LHR increased from 5.37 to 9.05; *p* = 0.0108); (ii) the EQ-VAS to the IPSS (LHR increased from 7.32 to 11.56; *p* = 0.0031) or the R-IPSS (LHR increased from 5.37 to 10.28; *p* = 0.0058); (iii) the EQ-5D-5L index to the IPSS (LHR increased from 7.32 to 13.02; *p* = 0.0015) or the R-IPSS (LHR increased from 5.37 to 13.48; *p* = 0.0012), indicating that they provided added value to the IPSS and R-IPSS (Table 4, grey shaded columns).

For the endpoint TTNT, significant increases of the LHR were observed after addition of (i) the LSS to the R-IPSS (LHR increased from 4.31 to 7.74; *p* = 0.0209); (ii) the EQ-VAS to the R-IPSS (LHR increased from 4.31 to 6.85; *p* = 0.0327); (iii) the EQ-5D-5L index to the IPSS (LHR increased from 3.60 to 6.38; *p* = 0.0411) or the R-IPSS (LHR increased from 4.31 to 6.55; *p* = 0.0378), indicating that they provided added value to the IPSS and R-IPSS (Table 4, grey shaded columns).

### 3.6. EQ-5D-5L Composite Scores at Azacitidine Start Impact Time-to-Event Endpoints

**Figure 3:**
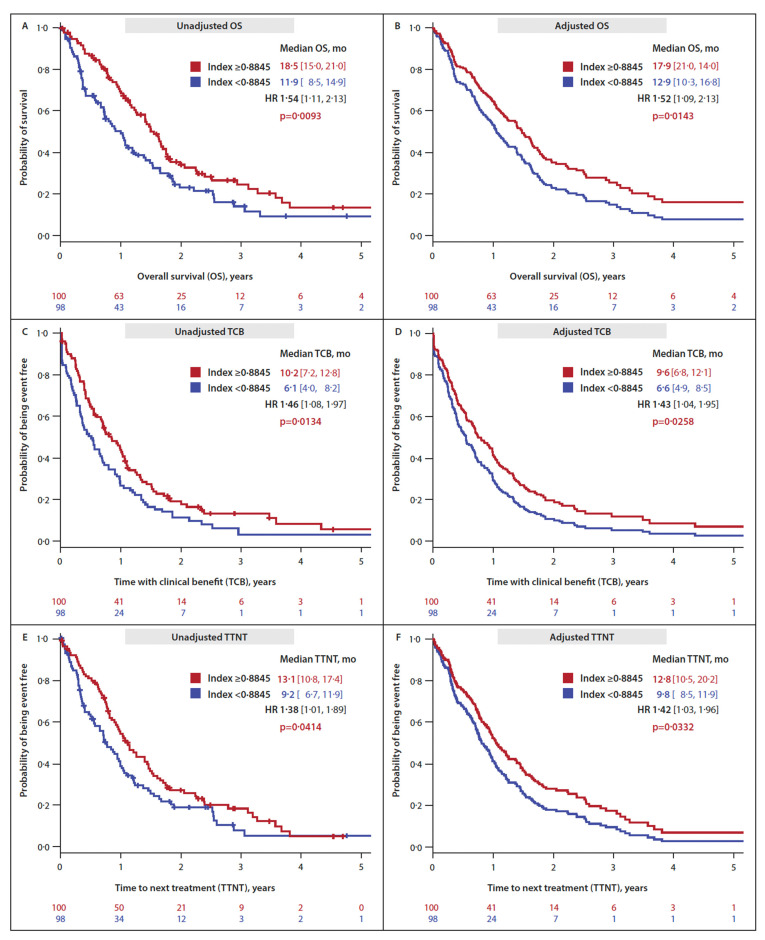
**Impact of the EQ-5D-5L index at azacitidine treatment start on time-to-event endpoints.** (**A**) Endpoint overall survival (OS), unadjusted. (**B**) Endpoint OS, adjusted ^1^. (**C**) Endpoint time with clinical benefit (TCB), unadjusted. (**D**) Endpoint TCB, adjusted ^1^. (**E**) Endpoint time to next treatment (TTNT), unadjusted. (**F**) Endpoint TTNT, adjusted ^1^. (^1^ Adjusted for the following characteristics at azacitidine treatment start: ECOG-PS, number of comorbidities, platelet count ≤30 G/L or platelet transfusion dependence, peripheral blood blasts, azacitidine treatment line and azacitidine dose in cycle 1).

Myeloid patients with an EQ-5D available at azacitidine treatment start (*n* = 205) were stratified according to their LSS, EQ-VAS or EQ-5D-5L index being </≥ the respective group median. In unadjusted analyses, patients with (i) an LSS < 8.0 at azacitidine treatment start had significantly longer OS and showed a trend for longer TCB and TTNT; (ii) an EQ-VAS < 65 at azacitidine treatment start had significantly longer OS; (iii) an EQ-5D-5L index ≥0.8845 had significantly longer OS, longer TCB and longer TTNT (Table 5, first four columns) (Figure 3A,C,E).

After multivariate adjustment (for ECOG-PS, number of comorbidities, platelet count ≤30 G/L or transfusion dependence, peripheral blood blasts, azacitidine treatment line and azacitidine dose in cycle one) patients with an EQ-5D-5L index above the group median (i.e., ≥0.8845) had significantly longer OS (17.9 months [95% CI 14.0–21.0] vs. 12.9 months [10.3–16.8]; HR 1.52 [1.09–2.13]; *p* = 0.0143), longer TCB (9.6 months [95% CI 6.8–12.1] vs. 6.6 months [4.9–8.5]; HR 1.43 [1.04–1.95]; *p* = 0.0258) and longer TTNT (12.8 months [95% CI 10.5–20.2] vs. 9.8 months [8.5–11.9]; HR 1.42 [1.03–1.96]; *p* = 0.0332) (Table 5, last three columns) (Figure 3B,D,F).

### 3.7. EQ-5D-5L Composite Scores at Azacitidine Start Prognosticate the Likelihood of Response to Azacitidine

In univariate logistic regression, the LSS (*p* = 0.0009), EQ-VAS (*p* = 0.0237) and EQ-5D-5L index (*p* = 0.0110) were significantly correlated with response to azacitidine. After multivariate adjustment, LSS remained significantly predictive of response to azacitidine (*p* = 0.0160; OR 0.451 [95% CI 0.235–0.852]), and the EQ-5D-5L index showed a trend (*p* = 0.0627; OR 0.522 [0.296–1.032]) (Table 6). An LSS of ≥8 at azacitidine treatment start thus indicates a significantly lower chance of responding to azacitidine as expressed by the OR of 0.45.

### 3.8. Longitudinal Assessment of EQ-5D-5L Responses and Clinical Parameters

**Figure 4:**
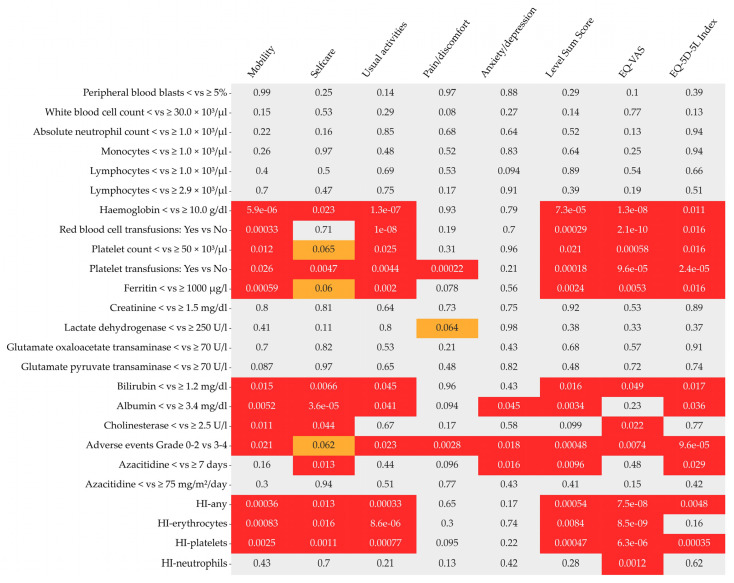
**Heatmap of *p*-values from multivariate-adjusted mixed-effect linear models of longitudinally assessed EQ-5D-5L response/clinical parameter pairs.** The individual boxes contain the *p*-values (red coloured *p*-values denote significant values ≤0.05, orange denotes a trend and is used for p-values between >0.05 and ≤0.065) of the corresponding multivariate-adjusted mixed-effect linear models using EQ-5D-5L responses as endogenous variables (*x*-axis), and various clinical measurements as exogenous variables (*y*-axis). Multivariate adjustment was performed by admitting the following variables remaining in the final Cox model as covariates: ECOG-PS, number of comorbidities, platelet count/platelet transfusion dependence, peripheral blood blasts, azacitidine treatment line and azacitidine dose in cycle one.

Multivariate-adjusted mixed-effect linear models of up to 1432 longitudinally assessed EQ-5D-5L response/dichotomised clinical parameter pairs revealed significant associations for haemoglobin level, red blood cell transfusion dependence, platelet count, platelet transfusion dependence, levels of ferritin, bilirubin, albumin, cholinesterase, the occurrence of adverse events, number of days with azacitidine treatment, and haematologic improvement (HI-any, HI-E, HI-P) with at least two EQ-5D dimensions, and at least one of the EQ-5D composite variables (LSS, EQ-VAS, EQ-5D-5L index) (Figure 4, Table 7). Sensitivity analyses for continuous clinical parameters yielded similar results (Supplementary pp. 17–18).

### 3.9. Minimally Clinically Important Differences

Of the statistically significant associations found in the dichotomised analyses, the following exhibited an effect size equal to or larger than the minimally clinically important difference: platelet transfusion dependence (LSS), ferritin ≥1000 µg/L (LSS), albumin ≥3.4 mg/dL (LSS), adverse events grade 3–4 (LSS, EQ-5D-5L index) and cholinesterase ≥2.5 U/L (EQ-VAS). These findings were corroborated in sensitivity analyses using continuous parameters.

## 4. Discussion

To our knowledge, our group is the first to compare EQ-5D-5L data of patients with MDS, CMML or AML with data from a reference population from a similar ethnic, socioeconomic and geographic background. In this prospective cohort analysis, we found that patients treated with azacitidine had significantly worse HRQoL than the German population norm (i.e., a representative sample of the German general adult population) [28,29] matched by sex, age group and number of comorbidities. In contrast to observations by Stauder et al. [10] who used the EQ-5D-3L, all significant differences observed for the EQ-5D-5L index and the EQ-VAS fulfilled the definitions of the minimally clinically important difference used by that group (>0.03 on the index and >3.0 on the EQ-VAS).

The current gold standards of prognostication in patients with MDS/CMML and low blast count AML are the International Prognostic Scoring System (IPSS) [26] and the revised IPSS (R-IPSS) [27]. The clinical relevance of these scores is underscored by the fact that approval of azacitidine for MDS patients in Europe is restricted to those with higher-risk IPSS (i.e., intermediate-2 and high risk categories). To our knowledge, our data are the first to indicate that LSS, EQ-VAS and EQ-5D-5L index at azacitidine treatment start provided added value to the IPSS and R-IPSS for the endpoints OS and TTNT. Other groups have prominently shown that patient-reported outcomes (other than EQ-5D) may predict OS and/or add value to the (R)-IPSS in elderly patients with MDS [30,31,32] or AML [21]. However, these questionnaires/indices incorporate 30 [30,32], 42 [31] and 44 items [21], many of which are not routinely assessed in patients with myeloid neoplasms, thus hampering the clinical everyday utility outside of clinical trials.

Our data are the only information on the impact of HRQoL, as assessed by the EQ-5D-5L, on time-to-event endpoints of patients treated with azacitidine. After multivariate adjustment (for ECOG-PS, number of comorbidities, platelet count ≤30 G/L or transfusion dependence, peripheral blood blasts, azacitidine treatment line and azacitidine dose in cycle one) an EQ-5D-5L index <0.8845 at azacitidine start indicated a significantly shorter median survival (−5.0 months), an increased risk of death (+52%), significantly shorter azacitidine treatment duration (−3.0 months), shorter TTNT or death (−3.0 months) and a significantly higher risk of requiring a next treatment or dying (+42%). Our data further show that an LSS of ≥8 at azacitidine start indicates a significantly lower chance of responding to the drug (OR 0.45).

This is the first report on the longitudinal assessment of EQ-5D-5L responses with clinical parameters. Multivariate adjusted mixed-effect linear modelling revealed significant associations for EQ-5D-5L response parameters with clinical parameters associated with haematologic improvement, disease progression, or the occurrence of adverse events. Thus, these data show that quality of life ameliorated in responding patients and deteriorates in patients experiencing disease progression or grade 3–4 adverse events. It is difficult to interpret these findings compared with the wider literature as longitudinal analyses of HRQoL data on patients with MDS, CMML or AML are scarce, performed with questionnaires other than EQ-5D-5L and are often without multivariate adjustment. Efficace et al. found no association between ferritin levels and HRQoL as assessed by EORTC QLQC30 both at baseline and during the study period in heavily transfused patients with MDS treated with iron chelation therapy using linear mixed-effect models [33]. We observed significant associations of ferritin, bilirubin and albumin levels with problems in six of eight EQ-5D-5L dimensions/composite variables. This is the first indication that these clinical variables, two of which (hypalbuminaemia and hyperferritinaemia) have been shown to be associated with adverse prognosis in patients with MDS [34,35,36], CMML [37] or AML [38,39] correlate with HRQoL.

Little is known of the longitudinal effect of azacitidine on patients’ HRQoL, but recent publications demonstrating significant improvements of EQ-VAS and/or EQ-5D-5L index in patients responding to treatment in other malignancies [40,41] highlight the contemporality and clinical relevance of the topic.

The mean (SD) EQ-VAS and EQ-5D-5L index values of our cohort were similar to those previously reported in patients with MDS/CMML or AML. Problems (slight, moderate, severe or extreme) were most commonly self-reported in the dimensions of usual activities (59%), mobility (51%), pain/discomfort (50%), anxiety/depression (49%) and selfcare (22%). Similar to the lower-risk MDS population reported by Stauder et al. [10], (i) MDS, CMML and AML patients in our cohort had the least problems in the dimension of selfcare, (ii) no correlation could be found between IPSS or R-IPSS risk group at azacitidine treatment start and EQ-5D responses, and (iii) patient-related factors such as haemoglobin <10 g/dL, red blood cell transfusion dependence, ECOG-PS ≥ 2 and high-risk HCT-CI were found to be associated with significantly more problems in several dimensions and/or significantly worse EQ-5D-5L composite variables. We could, however, not find a significant difference in EQ-5D-5L response by sex or age group.

A limitation of this study is that we cannot speculate what the HRQoL would have been without azacitidine therapy. Furthermore, this question cannot be addressed by real-world evidence or by future randomised clinical trials due to ethical reasons. A further limitation is that we do not have EQ-5D-5L questionnaires for all patients for all treatment cycles. However, to impose mandatory pre-specified required time-points for filling out EQ-5D-5L questionnaires would be against the non-interventional nature of non-interventional studies in general, and of the Austrian Registry of Hypomethylating Agents in particular. Furthermore, these results cannot, eo ipso, be generalised to other treatments of patients with MDS, CMML or AML, as we exclusively studied HRQoL of patients treated with azacitidine. In the future, we aim to analyse EQ-5D-5L responses in myeloid patients irrespective of treatment type within the Austrian Myeloid Registry (NCT04438889; Ethics committee approval was provided by the Ethikkommission für das Bundesland Salzburg (415-E/2581/Feb-2020)), which is a disease-specific (rather than a drug-specific) registry, once sufficient data have been accumulated, and are open for collaborations with other study groups in this regard.

The strengths of this study are that we report the first evidence-based data on all of the above; the prospective nature of data collection; the proven quality of our database in direct patient-level comparison with randomised phase-3 clinical trial data [23]; few missing data; calculation and validation of diagnosis, cytogenetic risk groups, and prognostic scores; response to reduce human errors; multivariate adjustment; longitudinal analyses; correction for multiple testing; and that additional sensitivity analyses confirmed the robustness of our results.

## 5. Conclusions

In conclusion, the current findings support the use of EQ-5D-5L instruments in future clinical trials and real-world evidence databases, in order to fully consider all factors that can be potentially associated with treatment outcomes. They also extend knowledge on the safety and efficacy of azacitidine by showing that clinical benefits such as improvement of laboratory values associated with haematologic improvement, as well as haematologic improvement itself, correlate with improved HRQoL.

## Figures and Tables

**Table 1:** Comparison of published EQ-VAS and EQ-5D index values in patients with MDS and AML.

First Author	Year Published	Patients, *n*	Disease	EQ-5D, Type	EQ-VAS, Mean (SD)	Index Value, Mean (SD)	Index Value, Median (IQR)	Impact on Time-to-Event Endpoint
**MDS**								
Szende A. [11]	2009	47	MDS	3L	NR	0.78 (NR)	NR	NR
Oliva E. [12]	2012	148	MDS	3L	60 (20)	NR	0.74 (0.62–0.85)	NR
Stauder R. [10]	2018	1683	Lower-risk MDS	3L	69.6 (20.1)	0.74 (0.23)	NR	NR
de Swart L. [9]	2020	NR	Lower-risk MDS	3L	70.5 (19.7)	NR	NR	EQ-5D-3L index was significantly associated with progression-free survival in univariate analysis
Pleyer L. (this article)	2023	162	MDS/CMML	5L	64.4 (21.2)	0.79 (0.3)	0.88 (0.73–0.95)	EQ-5D-5L index, LSS and EQ-VAS were significantly associated with overall survival and the likelihood to respond to azacitidine in univariate analysis; EQ-5D-5L index was significantly associated with overall survival, time with clinical benefit and time to next treatment in multivariate-adjusted analyses. LSS was significantly associated with the likelihood to respond to azacitidine in multivariate analysis.
**AML**								
Uyl-de Groot C.A. [13]	1998	NR NR	AML	3L	70.6 (NR) 64.8 (NR)	NR NR	NR NR	NR
Slovacek L. [14]	2007	NR	AML	3L	67.5 (NR)	NR	NR	NR
Leunis A. [15]	2014	88	AML	3L	74.6 (17.4)	0.82 (17.4)	NR	NR
Kurosowa S. [16]	2015	392	AML	3L	NR	NR	NR	NR
van Dongen-Leunis, A. [17]	2016	111	AML	5L	NR	0.81 (0.22)	0.87 (NR-NR)	NR
Mamolo C. [18]	2019	NR	AML	3L	61.2 (NR)	0.74 (NR)	NR	NR
Horvath Walsh L. [19]	2019	75	AML	3L	61.2 (NR)	0.74	NR	NR
Yu H. [20]	2020	NR/168 NR/168	AML	3L 5L	76.9 (15.1)	0.829 (0.16) 0.786 (0.25)	NR NR	NR
Peipert J. [21]	2020	307	AML	5L	61.9 (20.1)	0.67 (0.26)	NR	NR
Pratz K.W. [22]	2022	642	AML	5L	NR	NR	NR	NR
Pleyer L. (this article)	2023	110	AML	5L	64.7 (21.7)	0.83 (0.2)	0.89 (0.76–0.98)	EQ-5D-5L index, LSS and EQ-VAS were significantly associated with overall survival and the likelihood to respond to azacitidine in univariate analysis; EQ-5D-5L index was significantly associated with overall survival, time with clinical benefit and time to next treatment in multivariate-adjusted analyses. LSS was significantly associated with the likelihood to respond to azacitidine in multivariate analysis.

NR indicates not reported.

**Table 2:** Prevalence of problems in patients with myeloid neoplasias (assessed by EQ-5D-5L at azacitidine treatment start (*n* = 205) ^1^) by disease-related and patient-related parameters.

	Mobility Problem ^2^		Selfcare Problem ^2^		Usual Activities Problem ^2^		Pain/Discomfort Problem ^2^		Anxiety/Depression Problem ^2^		Level Sum Score ^3^			Index Value ^4^				EQ-VAS	
	*n*/*n* (%)	*p* ^5^	*n*/*n* (%)	*p* ^5^	*n*/*n* (%)	*p* ^5^	*n*/*n* (%)	*p* ^5^	*n*/*n* (%)	*p* ^5^	*n*	Mean (SD)	*p* ^5^	*n*	Mean (SD)	*p* ^6^	*n*	Mean (SD)	*p* ^6^
**Total cohort**																			
1st available EQ-5D	136/272 (50.0)	NA	68/72 (25.0)	NA	150/272 (55.1)	NA	138/272 (50.7)	NA	125/272 (46.0)	NA	266	9.1 (3.9)	NA	266	0.807 (0.232)	NA	263	63.9 (21.6)	NA
** **EQ-5D in cycle 1 or 2	104/205 (50.7)	NA	46/205 (22.4)	NA	120/205 (58.5)	NA	102/205 (49.8)	NA	100/205 (48.8)	NA	200	9.2 (3.9)	NA	198	0.810 (0.229)	NA	200	64.5 (21.4)	NA
**Disease-related parameters ^1^**																			
** **Azacitidine ≥2nd line: No ** **Yes	75/145 (52.1) 29/59 (49.2)	0.7045	35/143 (24.5) 11/59 (18.6)	0.3688	86/141 (61.0) 34/59 (557.6)	0.6577	76/143 (53.1) 26/59 (44.1)	0.2406	72/143 (50.3) 28/59 (47.5)	0.7085	141 59	9.3 (4.0) 8.7 (3.5)	0.3288	141 59	0.800 (0.243) 0.831 (0.192)	0.4282	141 57	63.3 (22.0) 67.5 (19.7)	0.2136
** **Diagnosis: MDS or CMML ** **AML	59/112 (52.7) 45/91 (49.5)	0.6472	28/111 (25.2) 18/91 (19.8)	0.3585	66/109 (60.6) 54/91 (59.3)	0.8619	66/111 (59.5) 36/91 (39.6)	0.0049	53/111 (47.4) 47/91 (51.6)	0.5812	109 91	9.4 (4.0) 8.8 (3.6)	0.2921	109 91	0.788 (0.256) 0.835 (0.192)	0.2160	110 88	64.4 (21.2) 64.7 (21.7)	0.9440
** **Treatment-related disease: No ** **Yes	89/175 (50.9) 12/24 (50.0)	0.9372	39/174 (22.4) 6/24 (25.0)	0.7769	102/172 (59.3) 14/24 (58.3)	0.9279	84/174 (48.3) 15/24 (62.5)	0.1914	79/174 (45.4) 17/24 (70.8)	0.0194	172 24	9.1 (3.9) 9.5 (3.7)	0.4741	172 24	0.810 (0.238) 0.809 (0.182)	0.4869	170 24	64.7 (21.8) 64.4 (19.9)	0.7998
** **IPSS: Low or intermediate-1 ** **Intermediate-2 or high	39/72 (54.2) 62/125 (49.6)	0.5369	17/71 (23.9) 27/125 (21.6)	0.7055	40/69 (58.0) 75/125 (60.0)	0.7830	39/71 (54.9) 58/125 (46.4)	0.2510	32/71 (45.1) 64/125 (51.2)	0.4093	69 125	9.3 (4.2) 8.9 (3.5)	0.7663	69 125	0.789 (0.274) 0.836 (0.169)	0.7950	70 122	65.6 (20.7) 64.6 (21.8)	0.6783
** **R-IPSS: Very low or low ** **Intermediate, poor, very poor	11/26 (42.3) 90/169 (53.3)	0.2984	6/26 (23.1) 39/168 (23.2)	0.9877	13/26 (50.0) 102/166 (61.4)	0.2682	15/26 (57.7) 82/168 (48.8)	0.3992	12/26 (46.2) 84/168 (50.0)	0.7151	26 166	9.3 (5.0) 9.1 (3.6)	0.5927	26 166	0.758 (0.369) 0.821 (0.188)	0.7551	25 165	64.6 (21.8) 64.7 (21.1)	0.9609
** **IPSS cytogenetic risk: good ** **Intermediate or poor	60/125 (48.0) 34/56 (60.7)	0.1135	29/124 (23.4) 14/56 (25.0)	0.8143	67/123 (54.5) 35/55 (63.6)	0.2534	62/124 (50.0) 29/56 (51.8)	0.8244	64/124 (50.0) 27/56 (48.2)	0.6729	123 55	9.0 (3.9) 9.5 (3.8)	0.2706	123 55	0.814 (0.228) 0.806 (0.216)	0.3255	122 54	65.7 (21.4) 64.1 (21.1)	0.6006
** **Peripheral blood blasts: <10% ** **≥10%	78/156 (50.0) 26/47 (55.3)	0.5225	34/155 (21.9) 12/47 (25.5)	0.6065	94/153 (61.4) 26/47 (55.3)	0.4539	83/155 (53.5) 19/47 (40.4)	0.1150	77/155 (49.7) 23/47 (48.9)	0.9291	153 47	9.3 (4.0) 8.8 (3.3)	0.7245	153 47	0.798 (0.246) 0.847 (0.162)	0.4270	153 45	64.8 (20.9) 63.8 (23.1)	0.7879
** **Monocytes: <10% ** **≥10%	56/121 (46.3) 44/75 (58.7)	0.0918	23/121 (19.0) 22/74 (29.7)	0.0846	61/119 (51.3) 52/74 (70.3)	0.0091	60/121 (49.6) 41/74 (55.4)	0.4301	52/121 (43.0) 43/74 (58.1)	0.0402	119 74	8.5 (3.5) 10.1 (4.3)	0.0053	119 74	0.850 (0.193) 0.752 (0.267)	0.0052	118 73	67.7 (19.8) 61.5 (22.5)	0.0626
** **Haemoglobin: <10.0 g/dL ** **≥10.0 g/dL	81/142 (57.0) 23/61 (37.7)	0.0115	37/141 (26.2) 9/61 (14.8)	0.0739	89/139 (64.0) 31/61 (50.8)	0.0792	73/141 (51.8) 29/61 (47.5)	0.5807	71/141 (50.4) 29/61 (47.5)	0.7135	139 61	9.5 (4.0) 8.3 (3.5)	0.0295	139 61	0.790 (0.242) 0.855 (0.191)	0.0429	137 61	62.8 (21.0) 68.5 (21.8)	0.0545
** **Red blood cell transfusions: ≤3 ** **>3	62/138 (44.9) 22/26 (84.6)	0.0002	31/137 (22.6) 8/26 (30.8)	0.3724	75/135 (55.6) 20/26 (76.9)	0.0425	73/137 (53.3) 16/26 (61.5)	0.4384	64/137 (46.7) 15/26 (57.7)	0.3045	135 26	9.0 (4.0) 9.9 (3.2)	0.0723	135 26	0.809 (0.247) 0.864 (0.163)	0.1412	134 25	65.6 (21.7) 55.2 (17.6)	0.0147
** **Platelet count: <100 G/L ** **≥100 G/L	36/65 (55.4) 68/138 (49.3)	0.4165	17/65 (26.2) 29/137 (21.2)	0.4299	39/63 (61.9) 81/137 (59.1)	0.7092	34/65 (52.3) 68/137 (49.6)	0.7226	30/65 (46.2) 70/137 (51.1)	0.5117	63 137	9.4 (4.0) 9.0 (3.8)	0.4665	61 137	0.797 (0.254) 0.815 (0.218)	0.4980	64 134	65.8 (19.9) 63.9 (22.1)	0.6100
**Patient-related parameters ^1^**																			
** **Sex male: No ** **Yes	45/81 (55.6) 59/122 (48.4)	0.3152	21/81 (25.9) 25/121 (20.7)	0.3819	49/79 (62.0) 71/121 (58.7)	0.6366	44/81 (54.3) 58/121 (47.9)	0.3735	47/81 (58.0) 53/121 (43.8)	0.0475	79 121	9.6 (4.0) 8.9 (3.7)	0.1644	79 121	0.786 (0.261) 0.825 (0.206)	0.2445	77 121	66.3 (21.9) 63.4 (21.1)	0.2408
** **Age ≥75 yrs: No ** **Yes	47/105 (44.8) 57/98 (58.2)	0.0563	19/104 (18.3) 27/89 (27.6)	0.1159	64/103 (62.1) 56/97 (57.7)	0.5252	44/104 (42.3) 59/98 (59.2)	0.0165	51/104 (49.0) 49/98 (50.0)	0.8913	103 97	8.7 (3.4) 9.6 (4.2)	0.2478	103 97	0.832 (0.191) 0.785 (0.263)	0.2429	103 95	66.9 (21.0) 60.0 (21.6)	0.1083
** **ECOG-PS: 0–1 ** **≥2	74/163 (45.4) 30/40 (75.0)	0.0008	26/162 (16.0) 20/40 (50.0)	<0.0001	87/160 (54.4) 33/40 (82.5)	0.0012	79/162 (48.8) 23/40 (57.5)	0.3224	70/162 (43.2) 30/40 (75.0)	0.0003	160 40	8.4 (3.4) 12.0 (4.3)	<0.0001	160 40	0.847 (0.185) 0.659 (0.315)	<0.0001	159 39	66.5 (20.8) 56.6 (22.3)	0.0092
** **HCT-CI: Low risk ** **Intermediate risk ** **High risk	31/77 (40.3) 33/65 (50.8) 40/61 (65.6)	0.0127	13/77 (16.9) 12/65 (18.5) 21/60 (35.0)	0.0259	40/75 (53.3) 36/65 (55.4) 44/60 (73.3)	0.0406	38/77 (49.4) 26/65 (40.0) 38/60 (63.3)	0.0324	36/77 (46.8) 30/65 (46.2) 34/60 (56.7)	0.4155	75 65 60	8.3 (3.3) 8.9 (3.7) 10.4 (4.4)	0.0133	75 65 60	0.849 (0.186) 0.822 (0.224) 0.748 (0.271)	0.0189	75 64 59	67.8 (20.1) 65.4 (21.0) 59.5 (22.7)	0.0750
** **No. of comorbidities: 0–1 ** **≥2	52/116 (44.8) 52/87 (59.8)	0.0350	23/116 (19.8) 23/86 (26.7)	0.2464	66/114 (57.9) 54/86 (62.8)	0.4841	57/116 (49.1) 45/86 (52.3)	0.6541	52/116 (44.8) 48/86 (55.8)	0.1225	114 86	8.7 (3.5) 9.8 (4.3)	0.0689	114 86	0.839 (0.183) 0.770 (0.276)	0.0703	113 85	66.8 (21.0) 61.6 (21.6)	0.0829

IPSS, International Prognostic Scoring System; IPSS-LR, IPSS lower-risk; IPSS-HR, IPSS higher-risk; R-IPSS, revised IPSS; ECOG-PS, Eastern Cooperative Oncology Group Performance Score; HCT-CI, Haematopoietic Stem Cell Comorbidity Index; MRC, Medical research Council. ^1^ EQ-5D in cycle 1 or 2 with a non-missing value for the respective parameter (hence patient numbers may vary slightly for each parameter analysed). ^2^ Problems were defined as answer options 2, 3, 4 or 5 for EQ-5D-5L and answer options 2 or 3 for EQ-5D-3L. ^3^ Represents the numerical sum of all EQ-5D responses. ^4^ The EQ-5D-5L index is measured on a scale from 0 to 1, whereby 0 indicates death and 1 perfect health. ^5^ Baseline parameters and EQ-5D-5L results were compared using the Chi-squared test (based on non-missing observations) for EQ-5D-5L problems (=2,3,4,5) vs. EQ-5D-5L no-problems (=1). ^6^ Baseline parameters and EQ-5D-5L results were compared using the Wilcoxon rank-sum test (also called Mann–Whitney U-test or Mann–Whitney–Wilcoxon Test) for Level Sum Score, EQ-5D-5L index value and EQ-VAS. Font color is red for all significant *p*-values <0.05.

**Table 3:** Comparison of HRQoL (as assessed by first available EQ-5D-5L) ^1^ between myeloid patients (*n* = 269) and a German population norm without myeloid neoplasias (*n* = 5001) ^2^ matched by age group, sex or number of comorbidities.

	Mobility Problem ^3^		Selfcare Problem ^3^		Usual Activities Problem ^3^		Pain/Discomfort Problem ^3^		Anxiety/Depression Problem ^3^		Index Value			EQ-VAS		
	*n*/*n* (%)	*p* ^4^	*n*/*n* (%)	*p* ^4^	*n*/*n* (%)	*p* ^4^	*n*/*n* (%)	*p* ^4^	*n*/*n* (%)	*p* ^4^	*n*	Mean (SD)	*p* ^5^	*n*	Mean (SD)	*p* ^5^
**Total cohort** Austrian Registry German Norm	136/269 (50.6) 1772/5001 (35.4)	<0.0001	68/268 (25.4) 360/5001 (7.2)	<0.0001	150/266 (56.4) 1417/5001 (28.3)	<0.0001	138/268 (51.5) 2847/5001 (56.9)	0.0802	125/269 (46.5) 1256/5001 (25.1)	<0.0001	266 5001	0.81 (0.23) 0.88 (0.18)	<0.0001	260 4997	63.9 (21.6) 71.6 (21.4)	<0.0001
**≥75 years** Austrian Registry German Norm	74/130 (56.9) 399/593 (67.3)	0.0245	39/130 (30.0) 111/593 (18.7)	0.0041	71/129 (55.0) 281/593 (47.4)	0.1151	75/130 (57.7) 418/593 (70.5)	0.0046	59/131 (45.0) 160/593 (27.0)	<0.0001	129 593	0.79 (0.25) 0.80 (0.28)	0.7547	127 590	61.7 (22.4) 60.9 (26.2)	0.7662
**65 < 75 years** Austrian Registry German Norm	50/105 (47.6) 324/654 (46.1)	0.7146	22/105 (21.0) 69/654 (10.6)	0.0023	60/104 (57.7) 198/654 (30.3)	<0.0001	49/105 (46.7) 411/654 (62.8)	0.0016	49/105 (46.7) 158/654 (24.2)	<0.0001	104 654	0.84 (0.19) 0.85 (0.240	0.5650	102 654	66.8 (19.3) 66.1 (25.5)	0.7777
**<65 years** Austrian Registry German Norm	12/34 (35.3) 1049/3754 (27.9)	0.3420	7/33 (21.2) 180/3754 (4.8)	<0.0001	19/33 (57.6) 938/3754 (25.0)	<0.0001	14/33 (42.4) 2017/3754 (53.7)	0.1948	17/33 (51.5) 938/3754 (25.0)	0.0005	33 3754	0.77 (0.26) 0.90 (0.15)	<0.0001	34 3753	63.5 (24.0) 74.2 (19.1)	0.0011
**Females** Austrian Registry German Norm	59/103 (57.3) 980/2584 (37.9)	<0.0001	30/103 (29.1) 203/2584 (7.9)	<0.0001	63/101 (62.4) 789/2584 (30.5)	<0.0001	60/103 (58.3) 1497/2584 (57.9)	0.9487	56/103 (54.5) 734/2584 (28.4)	<0.0001	101 2584	0.78 (0.26) 0.86 (0.20)	<0.0001	98 2581	64.6 (21.8) 71.1 (22.2)	0.0048
**Males** Austrian Registry German Norm	77/166 (46.4) 791/2417 (32.7)	0.0003	38/165 (23.0) 157/2417 (6.5)	<0.0001	86/165 (52.7) 628/2417 (26.0)	<0.0001	78/165 (47.3) 1350/2417 (55.9)	0.0319	69/166 (41.6) 522/2417 (21.6)	<0.0001	165 2417	0.83 (0.21) 0.90 (0.16)	<0.0001	165 2416	63.5 (21.5) 72.1 (20.5)	<0.0001
**One comorbidity** Austrian Registry German Norm	24/66 (36.4) 455/1432 (31.8)	0.4344	9/66 (13.6) 74/1432 (5.2)	0.0033	31/64 (48.4) 361/1433 (25.2)	<0.0001	32/66 (48.5) 813/1432 (56.8)	0.1843	31/67 (46.3) 317/1432 (22.1)	<0.0001	64 1432	0.87 (0.17) 0.90 (0.15)	0.0861	64 1432	66.3 (22.8) 73.0 (19.2)	0.0067
**Two comorbidities** Austrian Registry German Norm	42/85 (50.6) 378/820 (46.1)	0.4295	23/85 (27.1) 74/821 (9.0)	<0.0001	49/85 (57.7) 294/821 (35.8)	<0.0001	43/85 (50.6) 570/821 (69.4)	0.0004	31/85 (37.7) 245/821 (29.8)	0.1370	85 821	0.82 (0.21) 0.85 (0.18)	0.1154	83 821	65.7 (20.6) 65.1 (21.9)	0.7841
**≥Three comorbidities** Austrian Registry ** **German Norm	69/118 (58.5) 627/870 (72.1)	0.0024	36/117 (30.8) 179/871 (20.6)	0.0119	70/117 (59.8) 536/870 (61.6)	0.7104	63/117 (53.9) 748/871 (85.9)	<0.0001	62/117 (53.0) 374/871 (42.9)	0.0398	117 871	0.77 (0.27) 0.72 (0.28)	0.0944	116 871	61.3 (21.4) 55.2 (24.0)	0.0093

EQ-VAS indicates EuroQol Visual Analogue Scale. ^1^ First available EQ-5D with a non-missing value for the respective parameter (hence patient numbers may vary slightly for each parameter analysed). ^2^ Published and unpublished data provided by Grochtdreis et al. [28]. ^3^ Problems were defined as answer options 2, 3, 4 or 5 for EQ-5D-5L and answer options 2 or 3 for EQ-5D-3L. ^4^ The prevalence of EQ-5D-5L problems (=2,3,4,5) vs. EQ-5D-5L no-problems (=1) were compared using the Chi-squared test. ^5^ EQ-5D-Indices and EQ-VAS were compared between the Austrian Registry of Hypomethylating Agents and the German Norm cohorts using Student’s *T*-test. Font color is red for all significant *p*-values <0.05.

**Table 4:** Prognostic value of the IPSS and R-IPSS with or without baseline Level Sum Score (LSS), EQ Visual Analogue Scale (VAS) or EQ-5D-5l index value, by time-to-event endpoint (patients with EQ-5D-5L responses available at azacitidine treatment start (n = 205)).

	(R)-IPSS			(R)-IPSS + LSS	(R)-IPSS + EQ-VAS		(R)-IPSS + Index		
	Months [95% CI] ^1^	LHR	*p* ^6^	LHR	*p* ^6^	LHR	*p* ^6^	LHR	*p* ^6^
**Overall survival**									
IPSS: Lower-risk ^2^ Higher-risk ^3^	21.0 [14.6–30.3] 12.8 [10.2–16.9]	7.3195	0.0068	10.6911	0.0048	11.5552	0.0031	13.0219	0.0015
R-IPSS: Lower-risk ^4^ Higher-risk ^5^	30.3 [11.2–39.3] 14.6 [11.9–17.8]	5.3691	0.0205	9.0542	0.0108	10.2840	0.0058	13.4753	0.0012
**Time with clinical benefit**									
IPSS: Lower-risk ^2^ Higher-risk ^3^	8.9 [5.6–13.1] 7.9 [5.2–9.6]	1.0693	0.3011	3.6196	0.1637	1.9171	0.3835	3.6196	0.1637
R-IPSS: Lower-risk ^4^ Higher-risk ^5^	7.8 [3.4–14.9] 8.0 [6.4–9.6]	0.0757	0.7832	4.0208	0.1339	1.5603	0.4583	4.0208	0.1339
**Time to next treatment**									
IPSS: Lower-risk ^2^ Higher-risk ^3^	14.6 [9.5–19.3] 11.3 [8.9–12.6]	3.5998	0.0578	5.7236	0.0572	4.7933	0.0910	6.3834	0.0411
R-IPSS: Lower-risk ^4^ Higher-risk ^5^	17.6 [6.9–37.7] 10.8 [9.3–12.6]	4.3114	0.0379	7.7372	0.0209	6.8408	0.0327	6.5489	0.0378

IPSS, International Prognostic Scoring System; R-IPSS, revised IPSS; LHR, likelihood ratio test. ^1^ Estimated via univariate Cox proportional hazards regression. ^2^ IPSS lower-risk comprises IPSS low and intermediate-1 risk categories. ^3^ IPSS higher-risk comprises IPSS intermediate-2 and high risk categories. ^4^ R-IPSS lower-risk comprises R-IPSS very low and low risk categories. ^5^ R-IPSS higher-risk comprises R-IPSS intermediate, high and very high risk categories. ^6^ Estimated via multivariate Cox proportional hazards regression.

**Table 5:** Time-to-endpoint results for patients with EQ-5D-5L results available at azacitidine treatment start (*n* = 205).

	Univariate (*n* = 205)			Multivariate ^4^ (*n* = 205)		
	Months [95% CI]	*p*	HR [95% CI]	Months [95% CI]	*p*	HR [95% CI]
**Overall Survival**						
Level Sum Score: <median ^1^ ≥median	19.3 [14.6–21.5] 12.4 [8.7–15.0]	0.0407	1.408 [1.013–1.956]	16.9 [12.9–37.4] 14.2 [11.7–17.8]	0.2286	1.234 [0.876–1.737]
EQ-VAS (health today): ≥median ^2^ <median	17.9 [13.8–21.3] 12.8 [8.7–16.8]	0.0141	1.511 [1.084–2.106]	16.9 [12.9–30.6] 14.0 [11.4–24.7]	0.2293	1.242 [0.872–1.769]
EQ-5D-5L index: ≥median ^3^ <median	18.5 [15.0–21.0] 11.9 [8.5–14.9]	0.0093	1.536 [1.109–2.127]	17.9 [14.0–21.0] 12.9 [10.3–16.8]	0.0143	1.523 [1.088–2.131]
**Time with Clinical Benefit**						
Level Sum Score: <median ^1^ ≥median	10.2 [6.6–13.2] 6.1 [4.3–8.2]	0.0573	1.340 [0.989–1.815]	8.7 [6.5–11.8] 6.8 [5.2–8.8]	0.2174	1.221 [0.889–1.677]
EQ-VAS (health today): ≥median ^2^ <median	9.6 [6.6–12.1] 6.7 [4.6–8.5]	0.1841	1.227 [0.906–1.662]	8.4 [6.4–11.4] 7.7 [5.6–9.6]	0.5233	1.111 [0.998–1.012]
EQ-5D-5L index: ≥median ^3^ <median	10.2 [7.2–12.8] 6.1 [4.0–8.2]	0.0134	1.456 [1.078–1.966]	9.6 [6.8–12.1] 6.6 [4.9–8.5]	0.0258	1.425 [1.044–1.945]
**Time to Next Treatment**						
Level Sum Score: <median ^1^ ≥median	13.5 [9.8–17.6] 9.4 [7.6–11.9]	0.0633	1.347 [0.982–1.846]	12.6 [10.2–16.5] 10.8 [8.9–12.6]	0.1144	1.302 [0.938–1.806]
EQ-VAS (health today): ≥median ^2^ <median	12.6 [9.4–16.8] 11.1 [8.5–12.8]	0.1034	1.305 [0.946–1.801]	11.9 [9.7–14.6] 11.1 [9.0–20.2]	0.4197	1.150 [0.819–1.614]
EQ-5D-5L index: ≥median ^3^ <median	13.1 [10.8–17.4] 9.2 [6.7–11.9]	0.0414	1.383 [1.011–1.890]	12.8 [10.5–20.2] 9.8 [8.5–11.9]	0.0332	1.420 [1.028–1.962]

EQ-VAS indicates EuroQol Visual Analogue Scale. ^1^ Median for Level Sum Score: 8.0. ^2^ Median for EQ-VAS: 65. ^3^ Median for EQ-5D-5L index: 0.8845. ^4^ Adjusted for the covariates remaining in the final Cox model: ECOG-PS, number of comorbidities, platelet count/transfusion dependence, peripheral blood blasts, azacitidine treatment line and azacitidine dose in cycle one.

**Table 6:** Prognostic value of baseline Level Sum Score, EQ visual analogue scale (VAS) or EQ-5D-5L index value for the likelihood to respond to azacitidine (patients with EQ-5D-5L responses available at azacitidine treatment start (*n* = 205)).

	Univariate *p*	Multivariate ^4^ *p*	Multivariate ^4^ OR [95% CI]
Level Sum Score: ≥ vs. < median ^1^	0.0009	0.0160	0.451 [0.235–0.852]
EQ-VAS: < vs. ≥ median ^2^	0.0237	0.1065	0.590 [0.321–1.116]
EQ-5D-5L index: < vs. ≥ median ^3^	0.0110	0.0627	0.522 [0.296–1.032]

^1^ Median for Level Sum Score: 8.0. ^2^ Median for EQ-VAS: 65. ^3^ Median for EQ-5D-5L index: 0.8845. ^4^ Adjusted for the covariates remaining in the final Cox model: ECOG-PS, number of comorbidities, platelet count/transfusion dependence, peripheral blood blasts, azacitidine treatment line and azacitidine dose in cycle one.

**Table 7:** Multivariate-adjusted ^1^ longitudinal analyses of EQ-5D results and dichotomised parameters per azacitidine treatment cycle using mixed-effects linear models.

	Mobility		Selfcare		Usual Activities		Pain/Discomfort		Anxiety/Depression		Level Sum Score ^2^		EQ-VAS		EQ-5D-5L Index	
Differential blood count	*n* ^3^	*p*	*n*	*p*	*n*	*p*	*n*	*p*	*n*	*p*	*n*	*p*	*n*	*p*	*n*	*p*
** **Peripheral blood blasts< vs. ≥5%	1425	0.9897	1417	0.2548	1417	0.1447	1421	0.9703	1416	0.8775	1395	0.2930	1365	0.0996	1395	0.3916
White blood cell count< vs. ≥30.0 G/L	1429	0.1502	1421	0.5278	1421	0.2869	1425	0.0801	1420	0.2674	1399	0.1371	1368	0.7712	1399	0.1272
Absolute neutrophil count< vs. ≥1.0 G/L	1415	0.2206	1407	0.1586	1407	0.8529	1411	0.6784	1406	0.6362	1385	0.5171	1355	0.1329	1385	0.9389
Monocytes< vs. ≥1.0 G/L	1417	0.2559	1409	0.9738	1409	0.4770	1413	0.5203	1408	0.8287	1387	0.6366	1357	0.2476	1387	0.9439
Lymphocytes< vs. ≥1.0 G/L	1402	0.4021	1394	0.5043	1394	0.6879	1398	0.5349	1393	0.0941	1372	0.8871	1343	0.5429	1372	0.6557
Haemoglobin< vs. ≥10.0 g/dL	1429	<0.0001	1421	0.0227	1421	<0.0001	1425	0.9289	1420	0.7871	1399	<0.0001	1368	<0.0001	1399	0.0110
Red blood cell transfusions: Yes vs. No	1429	0.0003	1421	0.7072	1421	<0.0001	1425	0.1935	1420	0.6996	1399	0.0003	1368	<0.0001	1399	0.0161
Platelet count< vs. ≥50 G/L	1429	0.0122	1421	0.0647	1421	0.0248	1425	0.3142	1420	0.9574	1399	0.0212	1368	0.0006	1399	0.0156
Platelet transfusions: Yes vs. No	1429	0.0257	1421	0.0047	1421	0.0044	1425	0.0002	1420	0.2067	1399	0.0002	1368	<0.0001	1399	<0.0001
**Comorbidity/toxicity**																
Ferritin< vs. ≥1000 µg/L	723	0.0006	720	0.0598	720	0.0020	722	0.0785	718	0.5635	709	0.0024	703	0.0053	709	0.0163
Creatinine< vs. ≥1.5 mg/dL	1417	0.7976	1409	0.8133	1409	0.6386	1413	0.7286	1408	0.7550	1387	0.9162	1356	0.5338	1387	0.8874
Lactate dehydrogenase, U/L	1399	0.4066	1391	0.1095	1392	0.7977	1395	0.0642	1390	0.9778	1370	0.3834	1337	0.3343	1370	0.3673
Glutamate oxaloacetate transaminase, U/L	1406	0.7039	1398	0.8181	1399	0.5276	1402	0.2078	1397	0.4316	1377	0.6822	1345	0.5734	1377	0.9119
Glutamate pyruvate transaminase, U/L	1348	0.0867	1340	0.9662	1340	0.6501	1344	0.4822	1339	0.8201	1318	0.4770	1288	0.7212	1318	0.7369
Bilirubin< vs. ≥1.2 mg/dL	1407	0.0149	1399	0.0066	1399	0.0451	1403	0.9600	1398	0.4338	1377	0.0158	1346	0.0494	1377	0.0170
Albumin< vs. ≥3.4 mg/dL	583	0.0052	579	<0.0001	578	0.0412	580	0.0942	576	0.0454	567	0.0034	565	0.2309	567	0.0355
Cholinesterase< vs. ≥3.7 U/L	584	0.0108	581	0.0437	580	0.6728	582	0.1706	580	0.5751	567	0.0992	567	0.0216	567	0.7691
Adverse events ^4^ Grade 0–2 vs. 3–4	1429	0.0208	1421	0.0616	1421	0.0229	1425	0.0028	1420	0.0179	1399	0.0005	1368	0.0074	1399	<0.0001
**Azacitidine dose/regimen**																
Azacitidine< vs. ≥7 days	1429	0.1648	1421	0.0129	1421	0.4369	1425	0.0964	1420	0.0158	1399	0.0096	1368	0.4788	1399	0.0288
Azacitidine< vs. ≥75 mg/m^2^/day	1426	0.1485	1418	0.1155	1418	0.0249	1422	0.0168	1417	0.0001	1396	0.0003	1365	0.0040	1396	0.0013
**Haematologic improvement (HI)**																
HI-any ^5^: Yes vs. No	1275	0.0004	1268	0.0130	1270	0.0003	1272	0.6473	1266	0.1747	1248	0.0005	1221	<0.0001	1248	0.0048
HI-Erythrocytes: Yes vs. No	1296	0.0008	1289	0.0163	1291	<0.0001	1293	0.2981	1287	0.7419	1269	0.0084	1239	<0.0001	1269	0.1645
HI-Platelets: Yes vs. No	1317	0.0025	1310	0.0011	1311	0.0008	1315	0.0951	1310	0.2232	1288	0.0005	1262	<0.0001	1288	0.0003
HI-Neutrophils: Yes vs. No	1362	0.4299	1355	0.7016	1354	0.2083	1358	0.1326	1353	0.4239	1333	0.2837	1303	0.0012	1333	0.6162

^1^ Adjusted for the covariates remaining in the final Cox model: ECOG-PS, number of comorbidities, platelet count/transfusion dependence, peripheral blood blasts, azacitidine treatment line and azacitidine dose in cycle one. ^2^ Represents the numerical sum of all EQ-5D-5L responses. ^3^ Number of parameter/EQ-5D-5L response pairs. ^4^ Assessed according to CTCAEv4.0. ^5^ Includes HI-Neutrophils and/or HI-Erythrocytes and/or HI-Platelets. Font color is red for all significant *p*-values <0.05.

## Data Availability

The datasets supporting the conclusions of this article are included within the article and the Supplementary Materials. Data sharing of patient level data collected for the study is not planned. However, we are open to research questions asked by other researchers, and we are also open to data contributions by others. Participation requests or potential joint research proposals can be made at any timepoint to the corresponding author via email (dr.lisa.pleyer@gmail.com) and are subject to approval by the AGMT and its collaborators.

## Supplementary Materials

The following supporting information can be downloaded at https://www.mdpi.com/article/10.3390/cancers15051388/s1, Supplemental Table S1. Contributions of participating centres; Supplemental Table S2. Information on EQ-5D composite scores; Supplemental Table S3; Choice of appropriate value set region, value set and population norm; Supplemental Table S4. Further statistical details; Supplemental Table S5. Missing data; Supplemental Table S6. Univariate Cox regression analyses of baseline parameters present at azacitidine treatment start in patients with an available EQ-5D in cycle 1 or 2; Supplemental Table S7. Variables remaining in the final multivariate Cox model; Supplemental Table S8. Patient characteristics at azacitidine start; Supplemental Table S9. Lab values assessed at azacitidine start; Supplemental Table S10. Comorbidities assessed at azacitidine start; Supplemental Table S11. Azacitidine treatment and response characteristics; Supplemental Table S12. Most frequent response patterns of EQ-5D questionnaires at azacitidine treatment start; Supplemental Table S13. Most frequent response patterns of all EQ-5D questionnaires; Supplemental Table S14. Overview of EQ-5D responses by patient group and responder status; Supplemental Table S15. Multivariate-adjusted longitudinal analyses of EQ-5D-5L results and continuous parameters per azacitidine treatment cycle using mixed-effects linear models; Supplemental Figure S1. Heatmap of *p*-values resulting from multivariate-adjusted mixed-effect linear models of longitudinally assessed EQ-5D-5L responses and concomitantly assessed continuous clinical parameters; The Ethics Commottee Approval statement; The protocoll of the Austrian Registry of Hypomethylating Agents; The signed sponsor approval page; The informed consent of the Austrian Registry of Hypomethylating Agents. (References [42,43,44,45,46,47,48,49,50,51,52,53,54,55,56,57,58,59,60,61,62,63,64] are cited in the Supplementary Materials).
