# Supplementary material for: Health-Related Quality of Life as Assessed by the EQ-5D-5L Predicts Outcomes of Patients Treated with Azacitidine—A Prospective Cohort Study by the AGMT
Source: Cancers (Basel). 2023 Feb 22;15(5):1388. doi: 10.3390/cancers15051388 (PMC10000211; doi:10.3390/cancers15051388)
Supplement: Supplementary file 1 [file cancers-15-01388-s001.zip › cancers-2208597-supplementary.pdf]

Supplemental Table S1. Information on EQ-5D composite scores.

EuroQol Visual Analogue Scale (EQ-VAS)
• The EuroQol visual analogue scale (EQ-VAS) aims to capture a respondents self-reported perception and rating of their own overall current health on a scale from 0-100, whereby 100 is labelled as 'The best health you can imagine' and 0 as 'The worst health you can imagine'.
**Level Sum Score (LSS)**
• The Level Sum Score (LSS) summarizes an EQ-5D profile by simply adding up the numerical values of the responses (levels) of each of the five questions (i.e. dimensions), treating each levels label as if it were a number rather than a categorical description [1]. The responses 2 (Moderate problems…) of the 3L questionnaire and 3 (Moderate problems…) of the 5L questionnaire are identical, as are the responses 3 (Unable to…) of the 3L questionnaire and 5 (Unable to…) of the 5L questionnaire (please see sample questionnaires in English language in Chapter 4). Thus, the 3L responses were mapped to the 5L responses as follows: 1-> 1, 2 -> 3, and 3 -> 5, to be able to analyse both the 3L and 5L questionnaires conjointly.
**EQ-5D Index values**
• EQ-5D profile data can be supplemented by using a 'scoring' or 'weighting' system to convert profile data to a single number, the 'EQ-5D values', also called 'EQ-5D index' [1]. Responses to each dimension are recorded as single digit numbers (from 1-3 for the EQ-5D-3L and 1-5 for the EQ-5D-5L) used as labels to numerically describe the severity level selected in each question. The digits for the five dimensions can be combined to a 3- or 5-digit code that refers to one of 243 (3^5 in the case of EQ-5D-3L) or one of 3125 (5^5 in the case of EQ-5D-5L) possible unique health states. They have no numeric properties (i.e. one cannot assume that the state 21111 is better than the state 13111).[1] Therefore, an appropriate 'value set' or 'tariff' is needed to derive a 'summary index score'.<br>• An EQ-5D 'value set' is a collection of index values for all possible EQ-5D profiles or health states.[2] It is generated by standardized valuation procedures in which a representative sample of the general population of a country or region is asked to place a preference-based value on certain EQ-5D health states (i.e. to imagine living with various health problems).[2]<br>• EQ-5D-3L and EQ-5D-5L each have different value sets which provide weights for each of the health states according to the preferences of the general population of the specific country or region.[3] These quality of life weights or utilities are constructed to lie on a scale anchored by the value of 1, whereby 1 is 'Best imaginable health' and 0 is 'death'.[1] Of note, negative index values are also possible, which correspond to states valued to be 'worse than death' by the respondents.<br>• An internationally standardized valuation technology study protocol with improved methods for health state valuation and accompanying computer-based valuation software (the EuroQol-Valuation Technology (EQ-VT)) was developed by the EuroQol Group to create standard value sets for the EQ-5D-5L and to enable international comparability. EQ-VT prescribes the use of the composite time trade-off valuation technique complemented by a discrete-choice experiment. When linked to an appropriate value set (of the same or similar geographic region as the sample population), the responses to the EQ-5D dimensions can be converted to a single summary number called an 'index value'. The index value reflects how good or bad the respective unique health state is, according to the preferences of the general population of a country/region. Other terms for index value frequently used in the literature are 'preference weights', 'preference-based values', 'utilities' and 'QALY weights'. Comparative performance across groups of individuals (or patient groups) is driven by differences in the descriptive system and the associated country/region specific value sets.<br>• Of note, any 'value set' used to summarize EQ-5D data, can be expected to "introduce an exogenous source of variance into statistical inference".[4]

Supplemental Table S2. Comparison of EQ-VAS and EQ-5D index values in patients with MDS/AML.

First author	Year published	Patients, n	Disease	EQ-5D, type	EQ-VAS, Mean (SD)	Index value, Mean (SD)	Index value, Median (IQR)	Impact on time-to-event endpoint
**MDS**								
Szende A.[5]	2009	47	MDS	3L	NR	0.78 (NR)	NR	NR
Oliva E.[6]	2012	148	MDS	3L	60 (20)	NR	0.74 (0.62-0.85)	NR
Stauder R.[7]	2018	1683	Lower-risk MDS	3L	69.6 (20.1)	0.74 (0.23)	NR	NR
de Swart L.[8]	2020	NR	Lower-risk MDS	3L	70.5 (19.7)	NR	NR	EQ-5D-3L index was significantly associated with progression free survival in univariate analysis
Pleyer L.	2022	162	MDS/CMML	5L	64.4 (21.2)	0.79 (0.3)	0.88 (0.73-0.95)	EQ-5D-5L index, LSS and EQ-VAS were significantly associated with overall survival and the likelihood to respond to azacitidine in univariate analysis; EQ-5D-5L index was significantly associated with overall survival, time with clinical benefit and time to next treatment in multivariate-adjusted analyses. LSS was significantly associated with the likelihood to respond to azacitidine in multivariate analysis.
**AML**								
Uyl-de Groot C.A.[9]	1998	NR<br>NR	AML	3L	70.6 (NR)<br>64.8 (NR)	NR<br>NR	NR<br>NR	NR
Slovacek L.[10]	2007	NR	AML	3L	67.5 (NR)	NR	NR	NR
Leunis A.[11]	2014	88	AML	3L	74.6 (17.4)	0.82 (17.4)	NR	NR
Kurosowa S.[12]	2015	392	AML	3L	NR	NR	NR	NR
van Dongen-Leunis, A.[13]	2016	111	AML	5L	NR	0.81 (0.22)	0.87 (NR-NR)	NR
Mamolo C.[14]	2019	NR	AML	3L	61.2 (NR)	0.74 (NR)	NR	NR
Horvath Walsh L.[15]	2019	75	AML	3L	61.2 (NR)	0.74	NR	NR
Yu H.[16]	2020	NR/168<br>NR/168	AML	3L<br>5L	76.9 (15.1)	0.829 (0.16)<br>0.786 (0.25)	NR<br>NR	NR
Peipert J.[17]	2020	307	AML	5L	61.9 (20.1)	0.67 (0.26)	NR	NR
Pratz KW.[18]	2022	642	AML	5L	NR	NR	NR	NR
Pleyer L.	2022	110	AML	5L	64.7 (21.7)	0.83 (0.2)	0.89 (0.76-0.98)	EQ-5D-5L index, LSS and EQ-VAS were significantly associated with overall survival and the likelihood to respond to azacitidine in univariate analysis; EQ-5D-5L index was significantly associated with overall survival, time with clinical benefit and time to next treatment in multivariate-adjusted analyses. LSS was significantly associated with the likelihood to respond to azacitidine in multivariate analysis.

NR indicates not reported.

Supplemental Table S3. Contributions of participating centres.

Participating centres	Patients, n	EQ5D questionnaires, n	Cycles azacitidine, n	Questionnaires not evaluable, n[1]
Wilhelminen	2	2	84	0
Steyr	3	3	50	0
Krems	4	4	61	0
Leoben	4	5	39	0
Wels	29	109	310	0
Innsbruck	70	387	695	0
Salzburg	160	946	2900	6
TOTAL	272	1456	4139	6

[1]No questions answered (empty questionnaire).

Supplemental Table S4. Choice of appropriate value set region, value set and population norm.

Choice of value set region

- EuroQol Group's recommendation to select a value set that most closely approximates the region/country under investigation.[3,19,20]
- Several groups have concluded that country or region specific value-sets should be used whenever possible.[21,22 23]
- No EQ-5D value set exists for Austria, neither for 3L nor for 5L.
- Germany is a neighbouring country, where inhabitants speak the same language, have a similar standard of living, a similar political and social system, and a similar socioeconomic background. Hence, similar values may be assumed.

➔ **After prior consultation with EuroQol, it was decided to select a value set generated in Germany.**

Choice of crosswalk direction and of German value set

- Many more 5L questionnaires than 3L questionnaires were available.
- Comparative performance of the 5L versus the 3L suggests that the 5L is an improvement upon the 3L due to the observation of (i) reduced ceiling effects and of (ii) an enhanced discriminative capacity for the detection of distinctions.[24,25]
- The German cohort used to generate the 'EQ-5D-3L German value set' consisted of only n=339 individuals.[23]
- The data published by Greiner et al. was collected between 1997-1998 and is thus more than two decades old. It may no longer reflect the current preferences of the general German population.
- Modelling by Greiner et al. did not include the combined use of time trade-off and discrete-choice experiment methods, as suggested by EuroQol.
- In general, inconsistent study designs and methods were used to generate value sets for the EQ-5D-3L instrument.[26,27]
- The United Kingdom National Institute for Health and Care Excellence (NICE) recommends the use of an updated international standard protocol for generating value sets.[28]
- A German value set for the EQ-5D-5L was recently published in 2018, included n=1.158 participants and was the first to implement the most up-to-date internationally standardized EuroQol-Valuation Technology (EQ-VT) version 2.0.[29]
- EuroQol recommended the conversion of 3L to 5L data, i.a. because the report by Ludwig et al. used the standardized EQ-VT version 2.0.
- Each level of the EQ-5D-5L value set can be ascribed a certain utility weight that is consistent throughout (as opposed to the 3L value set).[29]

➔ **After prior consultation with EuroQol, it was decided to use the reverse-crosswalk tool kindly provided by EuroQol on November 16 2020, and the value set published by Ludwig et al for this study.[29]**

Choice of population norm

- No EQ-5D population norm data exist for Austria.
- Five publications of German population norms were published at the time of writing.
  - Huber et al. published data for the general German population using the EQ-5D-5L questionnaire but did not include EQ-5D-5L index values or provide data on patients stratified by numbers of comorbidities.[30]
  - König et al. used the EQ-5D-3L.[31]
  - Hinz et al. used the EQ-5D-3L.[32]
  - The data published for the general German population by Hinz et al.[33] several years later used EQ-5D-5L questionnaires, but applied a 3L value set using the crosswalk published by van Hout et al.[34] In addition, they included very few patients that were older than 60 years of age, and did not stratify their data by the number of comorbidities.[33]
  - Grochtdreis et al.[35] This publication is the first to use the German value set published by Ludwig et al.,[29] which in turn is the first published value set using the most up-to-date internationally standardized and recommended EQ-VT version 2.0.[28]

➔ **After prior consultation with EuroQol, it was decided to use the German population norm that was recently published by Grochtdreis et al.[35] The authors kindly provided additional data, that was not included in their publication.**

Minimally important differences (MID)

- To determine whether the thresholds for the dichotomised analysis yielded at least minimally important differences (MID), we compared the estimated model coefficients (which represent an estimated difference caused by clearing the threshold) in the linear mixed-effect models with existing guidelines for MID in the LSS (+/- 1.0), EQ-VAS (+/- 7 points),[36] and EQ-5D-5L index (+/-0.083).[37]

Supplemental Table S5. Further statistical details.

**Definition of outcomes**
• **Marrow response** was defined according to current response criteria for MDS/CMML[38] and AML[39] and was assessed at each bone marrow evaluation.<br>• **Haematologic improvement** was assessed according to the current International Working Group (IWG) Criteria 2006[38] (**appendix p5**). Haematologic improvement was assessed on day 1 of each azacitidine cycle for all patients.<br>• **Overall survival** was defined as the time from the first day of azacitidine treatment to death from any cause. Patients still alive or lost to follow-up were censored at last follow-up.<br>• **Time with clinical benefit** was defined as the time from the first day of azacitidine treatment to permanent treatment discontinuation due to any cause. Patients still alive or lost to follow-up and who had not terminated treatment with azacitidine were censored at last follow-up.<br>• **Time to next treatment** as surrogate for the cessation of treatment benefit was defined as the time from the first day of azacitidine treatment to the first day of the next (ensuing) treatment or death. Patients still alive or lost to follow-up and who had received no next treatment were censored at last follow-up.
**Cox regression models**
• **Cox regression models** for time-to-event endpoints were applied to identify and account for possible interrelations between predictors. Baseline characteristics with a predefined p-value of <0.1 in univariate Cox-regression for association with overall survival were entered into the multivariate Cox regression model (**appendix p7**). After stepwise selection, six variables remained in the final model (appendix p8) and were used for multivariate adjustment of Kaplan-Meier curves and linear mixed-effect modelling: ECOG-PS, number of comorbidities, platelet count ≤30 G/L or transfusion dependence, peripheral blood blasts, azacitidine treatment line, and azacitidine dose in cycle one.
**Linear mixed-effect modelling (LMM)**
• To identify variables that might be associated with patient reported outcomes, linear mixed-effect modelling was utilized, with patient identity as the grouping variable (a simple correlation analysis was not possible due to the data structure, typically incorporating several measurements per patient which cannot be assumed to be independent). Linear mixed-effect modelling allows the full incorporation of available longitudinal data. Responses from the EQ-5D questionnaire (as well as derived quantities like the LSS and the EQ-5D-5L index value) were used as endogenous variables, and various longitudinally assessed laboratory, response and toxicity measurements were used as exogenous variables. To summarize the linear mixed-effect modelling results, p-values were generated with respect to the null hypothesis that the true model parameter is zero. These were then visualised in heatmaps (both raw and with Benjamini-Yekutieli correction for multiple testing). Clinical parameters were dichotomised according to clinically relevant cut-offs.

Supplemental Table S6. Missing data.

Missing clinical data
• Missing data are listed in appendix pp9-14 and were mostly rare, i.e. between 0.0 and <5.0 %, except for certain laboratory values which are not routinely assessed, such as e.g. albumin.<br>• No imputations were performed for missing data. Only observed values were analysed.
**Missing EQ-5D data**
• In total 6 of 1456 (0.4%) questionnaires were excluded from the analyses (empty questionnaire), leaving a total of 1450 (100.0%) evaluable questionnaires in the study.<br>• In 85 of 1450 (5.0%) questionnaires EQ-VAS was not reported, and in 12 of 1450 (0.8%) questionnaires only the EQ-VAS was completed.<br>• 36 of 1450 (2.5%) questionnaires had missing responses in one of the five dimensions: mobility, selfcare, usual activities, pain/discomfort, or anxiety/depression.<br>• The Level Sum Score and EQ-5D Index were only calculated for patients with responses in all five dimensions, i.e. in 1402 (96.7%) of 1450 filled out questionnaires.<br>• 225 (15%) of 1456 EQ-5D-3L questionnaires were collected during January 2012 to June 2013; thereafter 1231 (85%) of EQ-5D-5L questionnaires were collected. The reverse crosswalk tool provided by EuroQol on November 16, 2020 was used to convert the 3L responses/indices to 5L responses/indices.<br>• No imputations were performed for missing data. Only observed values were analysed.

Supplemental Table S7. Univariate Cox regression analyses of baseline parameters present at azacitidine treatment start in patients with an available EQ-5D in cycle 1 or 2.

Variable	Observations, n	Events, n	Censored, n (%)	p
Age, yrs: continuous	205	150	55 (26.8)	0.1375
Sex: male/female	205	150	55 (26.8)	0.7005
Diagnosis at azacitidine start: MDS, CMML, AML	205	150	55 (26.8)	0.0576
ECOG-PS: 1, 2, 3, 4, or 5	205	150	55 (26.8)	0.0012
HCT-CI: low, intermediate, high	205	150	55 (26.8)	0.0168
Number of comorbidities: 0, 1, 2, or ≥3	205	150	55 (26.8)	0.0004
White blood cell count, G/L: continuous	205	150	55 (26.8)	0.3245
Haemoglobin ≤8.0 g/dL or red blood cell transfusion dependence: Yes vs No	205	150	55 (26.8)	0.1481
Platelet count ≤30 G/L or platelet transfusion dependence: Yes vs No	205	150	55 (26.8)	0.0389
Peripheral blood blasts, %: continuous	205	150	55 (26.8)	<.0001
Bone marrow blasts, %: continuous	205	150	55 (26.8)	0.0089
Azacitidine: 1st line or ≥2nd line	205	150	55 (26.8)	0.0123
Azacitidine dose in cycle 1, mg: continuous	205	150	55 (26.8)	0.0044
Azacitidine days in cycle 1, days: continuous	205	150	55 (26.8)	0.0139
Body mass index, kg/m^2: continuous	205	150	55 (26.8)	0.4873

ECOG-PS indicates Eastern Cooperative Oncology Group Performance Status; HCT-CI, Haematopoietic Cell Transplantation-specific Comorbidity Index.

Supplemental Table S8. Variables remaining in the final multivariate Cox model.

Variables entered into the model	Forward selection: Variables remaining	Backward selection: Variables remaining	Stepwise selection: Variables remaining
Diagnosis	Removed	Removed	Removed
ECOG-PS	0.0830	0.0833	0.0830
HCT-CI	Removed	Removed	Removed
Number of comorbidities, n	0.0006	0.0154	0.0006
Platelet count, G/L	0.0086	0.0054	0.0086
Peripheral blood blasts, %	<0.0001	0.0015	<0.0001
Bone marrow blasts, %	Removed	Removed	Removed
Azacitidine treatment line	0.0748	0.0650	0.0748
Azacitidine dose in cycle 1, mg/m^2	0.0124	0.0060	0.0124
Azacitidine days in cycle 1, n	Removed	Removed	Removed

ECOG-PS indicates Eastern Cooperative Oncology Group Performance Status; HCT-CI, Haematopoietic Cell Transplantation-specific Comorbidity Index.

Supplemental Table S9. Patient characteristics at azacitidine start.

	Patients with EQ-5D during azacitidine (n=272)	Patients with EQ-5D at azacitidine start (n=205)
Initial diagnosis: MDS, n (%)	144 (52.9)	108 (52.7)
CMML	32 (11.8)	21 (10.2)
AML[4]	89 (32.7)	71 (34.6)
CMPD	6 (2.2)	5 (2.4)
Unknown	1 (0.4)	0 (0.0)
Mean year of initial diagnosis (SD), years	2014.4 (3.7)	2014.9 (3.2)
Median (IQR)	2015.0 (2013.0-2017.0)	2015.0 (2013.0-2017.0)
Min-max	1990-2019	1997-2019
Unknown, n (%)	0 (0.0)	0 (0.0)
Diagnosis at azacitidine start: MDS, n (%)	129 (47.4)	95 (46.3)
CMML	33 (12.1)	19 (9.3)
AML[1]	110 (40.4)	91 (44.4)
Unknown	0 (0.0)	0 (0.0)
Mean year of azacitidine start (SD), years	2015.6 (2.5)	2016.0 (2.1)
Median (IQR)	2016.0 (2014.0-2018.0)	2016.0 (2014.0-2018.0)
Min-max	2007-2020	2012-2020
Unknown, n (%)	0 (0.0)	0 (0.0)
Mean age (SD), years	73.4 (8.8)	73.5 (8.4)
Median (IQR)	74.0 (69.0-79.0)	74.0 (69.0-79.0)
Min-max	24.0-93.0	45.0-93.0
≥ 75 years, n (%)	133 (48.9)	100 (48.8)
Unknown	0 (0.0)	0 (0.0)
Sex: Female, n (%)	104 (38.2)	82 (40.0)
Male	168 (61.8)	123 (60.0)
Unknown	0 (0.0)	0 (0.0)
ECOG-PS: 0-1, n (%)	221 (81.3)	164 (80.0)
2-4	51 (18.8)	41 (20.0)
Unknown	0 (0.0)	0 (0.0)
HCT-CI: Low, n (%)	100 (36.8)	77 (37.6)
Intermediate	90 (33.1)	65 (31.7)
High	82 (30.1)	63 (30.7)
Unknown	0 (0.0)	0 (0.0)
Treatment-related disease: No, n (%)	234 (86.0)	176 (85.9)
Yes	33 (12.1)	25 (12.2)
Unknown	5 (1.8)	4 (2.0)
MRC cytogenetic risk: Good, n (%)	5 (1.8)	2 (1.0)
Intermediate	176 (64.7)	131 (63.9)
Poor	63 (23.2)	49 (23.9)
Unknown	28 (10.3)	23 (11.2)
IPSS cytogenetic risk: Good, n (%)	173 (63.6)	126 (61.5)
Intermediate	40 (14.7)	31 (15.1)
Poor	31 (11.4)	25 (12.2)
Unknown	28 (10.3)	23 (11.2)
R-IPSS cytogenetic risk: Very good, n (%)	2 (0.7)	2 (1.0)
Good	173 (63.6)	126 (61.5)
Intermediate	44 (16.2)	34 (16.6)
Poor	23 (8.5)	18 (8.8)
Very poor	2 (0.7)	2 (1.0)
Unknown	28 (10.3)	23 (11.2)
IPSS lower-risk,[2] n (%)	103 (37.9)	73 (35.6)
IPSS higher-risk	159 (58.5)	125 (61.0)
Unknown	10 (3.7)	7 (3.4)
R-IPSS lower-risk,[3] n (%)	40 (14.7)	26 (12.7)
R-IPSS higher-risk	222 (81.6)	171 (83.4)
Unknown	10 (3.7)	8 (3.9)
Bone marow blasts, %: Mean (SD)	23.0 (26.2)	24.8 (26.9)
Median (IQR)	12.0 (5.0-35.0)	14.5 (5.0-37.0)
Min-Max	0.0-96.0	0.0-96.0
Unknown, n (%)	11 (4.1)	9 (4.4)

CMPD indicates chronic myeloproliferative diseases; ECOG-PS, Eastern Cooperative Oncology Group Performance Status; HCT-CI, Haematopoietic Cell Transplantation-specific Comorbidity Index.

MRC, Medical Research Council; IPSS indicates International Prognostic Scoring System; R-IPSS, revised IPSS.

[1]As defined by WHO-2016: ≥20% bone marrow or peripheral blood blasts.
[2]IPSS lower-risk comprises IPSS low and intermediate-1 risk categories.
[3]R-IPSS lower-risk comprises R-IPSS very low and low risk categories.

Supplemental Table S10. Lab values assessed at azacitidine start.

	Patients with EQ-5D during azacitidine (n=272)	Patients with EQ-5D at azacitidine start (n=205)
Peripheral blood blasts, %: Mean (SD)	9.7 (20.7)	11.0 (22.0)
Median (IQR)	1.0 (0.0-7.0)	1.0 (0.0-7.0)
Min-Max	0.0-98.0	0.0-98.0
Unknown, n (%)	0 (0.0)	0 (0.0)
White blood cell count, G/L: Mean (SD)	9.3 (15.2)	9.5 (15.6)
Median (IQR)	3.4 (1.9-8.2)	3.2 (1.9 / 8.8)
Min-Max	0.6-120.0	0.6-120.0
Unknown, n (%)	0 (0.0)	0 (0.0)
Absolute neutrophil count, G/L: Mean (SD)	3.6 (6.3)	3.5 (6.0)
Median (IQR)	1.3 (0.5-3.8)	1.2 (0.4-3.5)
Min-Max	0.0-50.8	0.0-49.0
Unknown, n (%)	8 (2.9)	7 (3.4)
Neutropenia (ANC <1.0 G/L): No, n (%)	158 (58.1)	119 (58.0)
Yes	114 (41.9)	86 (42.0)
Unknown	0 (0.0)	0 (0.0)
Monocytes, %: Mean (SD)	12.0 (13.5)	11.3 (13.3)
Median (IQR)	7.0 (2.0-17.0)	7.0 (2.0-15.0)
Min-Max	0.0-77.0	0.0-77.0
Unknown, n (%)	8 (2.9)	7 (3.4)
Lymphocytes, G/L: Mean (SD)	2.6 (6.9)	2.5 (7.1)
Median (IQR)	1.1 (0.8-1.8)	1.0 (0.8-1.7)
Min-Max	0.0-74.0	0.0-74.0
Unknown, n (%)	13 (4.8)	9 (4.4)
Haemoglobin, g/dL: Mean (SD)	9.3 (1.8)	9.3 (1.8)
Median (IQR)	9.0 (8.1-10.0)	8.9 (8.0-10.2)
Min-Max	4.8-14.8	5.9-14.5
Unknown, n (%)	0 (0.0)	0 (0.0)
Mean corpuscular volume, fl: Mean (SD)	92.6 (10.6)	92.2 (11.0)
Median (IQR)	92.0 (87.0-99.0)	92.0 (87.0-98.0)
Min-Max	27.0-120.0	27.0-116.0
Unknown, n (%)	1 (0.4)	1 (0.5)
Mean corpuscular haemoglobin, pg: Mean (SD)	31.3 (3.6)	31.3 (3.6)
Median (IQR)	31.0 (29.0-34.0)	31.0 (29.0-34.0)
Min-Max	19.0-41.0	19.0-40.0
Unknown, n (%)	1 (0.4)	1 (0.5)
Anaemia (Hb <11.0 g/dL): No, n (%)	45 (16.5)	37 (18.0)
Yes	227 (83.5)	168 (82.0)
Unknown	0 (0.0)	0 (0.0)
Red blood cell transfusion dependence: No, n (%)	186 (68.4)	147 (71.7)
Yes	86 (31.6)	58 (28.3)
Unknown	0 (0.0)	0 (0.0)
Platelet count, G/L: Mean (SD)	100.8 (111.1)	89.4 (82.3)
Median (IQR)	65.0 (32.0-126.5)	65.0 (32.0-124.0)
Min-Max	1.0-802.0	6.0-544.0
Unknown, n (%)	0 (0.0)	0 (0.0)
Thrombocytopenia (platelet count <100 G/L): No, n (%)	92 (33.8)	65 (31.7)
Yes	180 (66.2)	140 (68.3)
Unknown	0 (0.0)	0 (0.0)
Platelet transfusion dependence: No, n (%)	237 (87.1)	180 (87.8)
Yes	35 (12.9)	25 (12.2)
Unknown	0 (0.0)	0 (0.0)
Ferritin, µg/L: Mean (SD)	1065.9 (1239.3)	1062.7 (1342.8)
Median (IQR)	679.0 (361.0-1395.0)	612.5 (337.5-1386.5)
Min-Max	10.0-9198	10.0-9198
Unknown, n (%)	106 (39.0)	89 (43.4)
Lactate dehydrogenase, U/L: Mean (SD)	287.3 (171.1)	296.5 (183.1)
Median (IQR)	238.5 (183.0-332.0)	243.0 (187.0-337.0)
Min-Max	78.0-1306	78.0-1306
Unknown, n (%)	6 (2.2)	6 (2.9)
Cholinesterase, U/L: Mean (SD)	5.2 (1.8)	5.1 (1.8)
Median (IQR)	5.2 (3.9-6.4)	4.9 (3.7-6.3)
Min-Max	0.3-11.2	0.3-11.2
Unknown, n (%)	111 (40.8)	95 (46.3)
Glutamate oxaloacetate transaminase, U/L: Mean (SD)	28.7 (17.0)	29.5 (17.3)
Median (IQR)	24.5 (19.0-31.0)	26.0 (20.0-32.0)
Min-Max	9.0-137.0	9.0-137.0
Unknown, n (%)	6 (2.2)	4 (2.0)
Glutamate pyruvate transaminase, U/L: Mean (SD)	28.2 (27.6)	28.5 (26.4)
Median (IQR)	20.0 (14.0-29.0)	21.0 (15.0-30.0)
Min-Max	2.0-199.0	2.0-199.0
Unknown, n (%)	13 (4.8)	8 (3.9)

Creatinine, mg/dL: Mean (SD)	1.1 (0.6)	1.1 (0.7)
Median (IQR)	1.0 (0.8-1.2)	1.0 (0.8-1.2)
Min-Max	0.4-7.1	0.4-7.1
Unknown, n (%)	3 (1.1)	2 (1.0)
Bilirubin, mg/dL: Mean (SD)	0.7 (0.7)	0.7 (0.7)
Median (IQR)	0.6 (0.4-0.8)	0.6 (0.4-0.8)
Min-Max	0.1-7.2	0.1-7.2
Unknown, n (%)	6 (2.2)	5 (2.4)
Albumin, g/dL: Mean (SD)	3.8 (0.6)	3.8 (0.7)
Median (IQR)	3.9 (3.5-4.1)	3.8 (3.5-4.1)
Min-Max	1.2-6.3	1.2-6.3
Unknown, n (%)	123 (45.2)	105 (51.2)
Serum erythropoietin: < 50 IU/L, n (%)	32 (11.8)	20 (9.8)
50 < 500	57 (21.0)	45 (22.0)
≥ 500	12 (4.4)	4 (2.0)
Unknown	167 (61.4)	133 (64.9)

Supplemental Table S11. Comorbidities assessed at azacitidine start.

	Patients with EQ-5D during azacitidine (n=272)	Patients with EQ-5D at azacitidine start (n=205)
Comorbidities: 0, n (%)	67 (24.6)	51 (24.9)
1	85 (31.3)	65 (31.7)
2	61 (22.4)	44 (21.5)
3	33 (12.1)	28 (13.7)
4	20 (7.4)	13 (6.3)
5	4 (1.5)	2 (1.0)
6	2 (0.7)	2 (1.0)
Unknown	0 (0.0)	0 (0.0)
Body mass index, kg/m^2: <18.5, n (%)	8 (2.9)	7 (3.4)
18.5 <25	142 (52.2)	109 (53.2)
25 <30	87 (32.0)	64 (31.2)
30 <35	28 (10.3)	22 (10.7)
≥35	7 (2.6)	3 (1.5)
Unknown	0 (0.0)	0 (0.0)
Thromboembolism: No, n (%)	246 (90.4)	186 (90.7)
Yes	26 (9.6)	19 (9.3)
Unknown	0 (0.0)	0 (0.0)
Renal comorbidity: No, n (%)	221 (81.3)	165 (80.5)
Yes	51 (18.8)	40 (19.5)
Unknown	0 (0.0)	0 (0.0)
Hepatic comorbidity: No, n (%)	251 (92.3)	191 (93.2)
Yes	21 (7.7)	14 (6.8)
Unknown	0 (0.0)	0 (0.0)
Diabetes mellitus: No, n (%)	235 (86.4)	179 (87.3)
Yes	37 (13.6)	26 (12.7)
Unknown	0 (0.0)	0 (0.0)
Solid tumor: No, n (%)	223 (82.0)	165 (80.5)
Yes	49 (18.0)	40 (19.5)
Unknown	0 (0.0)	0 (0.0)
Active infection: No, n (%)	258 (94.9)	194 (94.6)
Yes	14 (5.1)	11 (5.4)
Unknown	0 (0.0)	0 (0.0)
Pulmonary comorbidity: No, n (%)	243 (89.3)	183 (89.3)
Yes	29 (10.7)	22 (10.7)
Unknown	0 (0.0)	0 (0.0)
Cardiac comorbidity: No, n (%)	187 (68.8)	140 (68.3)
Yes	85 (31.3)	65 (31.7)
Unknown	0 (0.0)	0 (0.0)
Additional haematologic neoplasm: No, n (%)	241 (88.6)	185 (90.2)
Yes	31 (11.4)	20 (9.8)
Unknown	0 (0.0)	0 (0.0)
Cerebrovascular disease: No, n (%)	250 (91.9)	190 (92.7)
Yes	22 (8.1)	15 (7.3)
Unknown	0 (0.0)	0 (0.0)
Rheumatologic comorbidity: No, n (%)	263 (96.7)	199 (97.1)
Yes	9 (3.3)	6 (2.9)
Unknown	0 (0.0)	0 (0.0)
Peptic ulcer: No, n (%)	265 (97.4)	200 (97.6)
Yes	7 (2.6)	5 (2.4)
Unknown	0 (0.0)	0 (0.0)
Inflammatory bowel disease: No, n (%)	270 (99.3)	203 (99.0)
Yes	2 (0.7)	2 (1.0)
Unknown	0 (0.0)	0 (0.0)
Psychiatric disturbance: No, n (%)	250 (91.9)	187 (91.2)
Yes	22 (8.1)	18 (8.8)
Unknown	0 (0.0)	0 (0.0)

Supplemental Table S12. Azacitidine treatment and response characteristics.

	Patients with EQ-5D during azacitidine (n=272)	Patients with EQ-5D at azacitidine start (n=205)
Mean year of azacitidine start (SD), years	2015.6 (2.5)	2016.0 (2.1)
Median (IQR)	2016.0 (2014.0-2018.0)	2016.0 (2014.0-2018.0)
Min-max	2007-2020	2012-2020
Unknown, n (%)	0 (0.0)	0 (0.0)
Status at last follow-up: Dead, n (%)	204 (75.0)	150 (73.2)
Alive and still on treatment with azacitidine	39 (14.3)	28 (13.7)
Alive and no longer treated with azacitidine	27 (9.9)	25 (12.2)
Lost to follow-up	2 (0.7)	2 (1.0)
Follow-up time from initial diagnosis, months: Mean (SD)	35.2 (39.1)	30.8 (33.7)
Median (IQR)	23.4 (12.3-40.9)	21.6 (11.0-35.3)
Min-Max	1.6-341.0	1.6-238.0
Unknown, n (%)	0 (0.0)	0 (0.0)
Follow-up time from azacitidine start, months: Mean (SD)	20.7 (20.2)	16.8 (15.7)
Median (IQR)	14.7 (7.8-26.7)	12.8 (6.2-21.8)
Min-Max	0.2-122.1	0.2-95.1
Unknown, n (%)	0 (0.0)	0 (0.0)
First cytopenias to azacitidine start, months: Mean (SD)	23.3 (38.0)	23.9 (39.0)
Median (IQR)	4.2 (1.4-27.5)	4.0 (1.3-26.6)
Min-Max	0.1-173.2	0.1-172.4
Unknown, n (%)	74 (27.2)	48 (23.4)
Initial diagnosis to azacitidine start, months: Mean (SD)	14.5 (34.1)	14.0 (31.0)
Median (IQR)	2.4 (0.9-14.0)	2.5 (0.9-14.0)
Min-Max	0.0-324.3	0.0-233.3
Unknown, n (%)	0 (0.0)	0 (0.0)
Transformation to AML: During azacitidine n (%)	33 (12.1)	26 (12.7)
After azacitidine	13 (4.8)	7 (3.4)
AML at azacitidine start	104 (38.2)	88 (42.9)
No transformation	122 (44.9)	84 (41.0)
Unknown	0 (0.0)	0 (0.0)
Time from azacitidine start to AML[1], months: Mean (SD)	21.7 (22.7)	17.2 (17.8)
Median (IQR)	14.6 (6.7-27.6)	10.6 (4.7-22.8)
Min-Max	0.0-122.1	0.0-95.1
AML at azacitidine start, n (%)	104 (38.2)	88 (42.9)
Unknown, n (%)	0 (0.0)	0 (0.0)
Duration of azacitidine treatment, months: Mean (SD)	14.6 (17.2)	10.9 (11.7)
Median (IQR)	9.1 (3.9-18.5)	7.7 (3.1-14.9)
Min-Max	0.1-102.2	0.1-73.5
Unknown, n (%)	0 (0.0)	0 (0.0)
First response type: Complete response (CR), n (%)	4 (1.5)	2 (1.0)
Incomplete CR (CRi)	9 (3.3)	6 (2.9)
Morphologic leukaemia free state (MLFS)	14 (5.1)	9 (4.4)
Partial response (PR)	0 (0.0)	0 (0.0)
Haematologic improvement (HI)	134 (49.3)	87 (42.4)
No response	111 (40.8)	101 (49.3)
Unknown	0 (0.0)	0 (0.0)
Time to 1st response, months: Mean (SD)	3.8 (3.7)	3.5 (3.2)
Median (IQR)	2.9 (1.8-4.1)	2.8 (1.8-3.9)
Min-Max	0.08-29.7	0.8-24.6
No response, n (%)	111 (40.8)	101 (49.3)
Unknown, n (%)	0 (0.0)	0(0.0)
Best response type: Complete response (CR), n (%)	35 (12.9)	17 (8.3)
Incomplete CR (CRi)	20 (7.4)	12 (5.9)
Morphologic leukaemia free state (MLFS)	18 (6.6)	15 (7.3)
Partial response (PR)	3 (1.1)	1 (0.5)
Haematologic improvement (HI)	85 (31.3)	59 (28.8)
No response	111 (40.8)	101 (49.3)
Unknown	0 (0.0)	0 (0.0)
Time to best response, months: Mean (SD)	5.5 (4.7)	5.0 (4.0)
Median (IQR)	4.5 (2.8-6.9)	3.8 (2.5-6.1)
Min-Max	0.8-30.4	0.8-24.6
No response	111 (40.8)	101 (49.3)
Unknown	0 (0.0)	0 (0.0)
Time with clinical benefit, months: Mean (SD)	14.7 (17.3)	10.9 (11.8)
Median (IQR)	9.3 (3.9-18.5)	7.7 (3.1-14.9)
Min-Max	0.1-102.2	0.1-74.6
Unknown, n (%)	0 (0.0)	0 (0.0)
Time to next treatment (TTNT) or death, months: Mean (SD)	17.9 (18.2)	13.7 (12.2)
Median (IQR)	12.5 (6.1-22.7)	10.2 (4.6-18.6)
Min-Max	0.2-111.1	0.2-71.3
No next treatment and dead, n (%)	143 (52.6)	104 (50.7)
No next treatment and alive, n (%)	52 (19.1)	41 (20.0)

Time from azacitidine start to death or last FU, months: Mean (SD)	20.7 (20.2)	16.8 (15.7)
Median (IQR)	14.7 (7.8-26.7)	12.8 (6.2-21.8)
Min-Max	0.2-122.1	0.2-95.1
Unknown, n (%)	0 (0.0)	0 (0.0)
Time from azacitidine stop to death, months: Mean (SD)	5.2 (7.5)	4.8 (7.0)
Median (IQR)	2.2 (0.9-6.9)	2.1 (0.9-6.0)
Min-Max	0.0-46.8	0.0-46.8
Dead at data cut-off (observations used), n (%)	204 (75.0)	150 (73.2)
Death within 30 days after azacitidine start: Yes, n (%)	7 (2.6)	7 (3.4)
No	265 (97.4)	198 (96.6)
Unknown	0 (0.0)	0 (0.0)
Death within 60 days after azacitidine start: Yes, n (%)	13 (4.8)	13 (6.3)
No	259 (95.2)	192 (93.7)
Unknown	0 (0.0)	0 (0.0)
1 year survival after azacitidine start: Yes, n (%)	184 (67.6)	126 (61.5)
No	88 (32.4)	79 (38.5)
Unknown	0 (0.0)	0 (0.0)
2 year survival after azacitidine start: Yes, n (%)	114 (41.9)	75 (36.6)
No	158 (58.1)	130 (63.4)
Unknown	0 (0.0)	0 (0.0)
3 year survival after azacitidine start: Yes, n (%)	88 (32.4)	63 (30.7)
No	184 (67.6)	142 (69.3)
Unknown	0 (0.0)	0 (0.0)
4 year survival after azacitidine start: Yes, n (%)	76 (27.9)	56 (27.3)
No	196 (72.1)	149 (72.7)
Unknown	0 (0.0)	0 (0.0)

[1]For patients with a diagnosis of MDS or CMML at azacitidine start.

Supplemental Table S13. Most frequent[1] response patterns of EQ-5D questionnaires[2] at azacitidine treatment start.[3]

Pattern	n	%	Pattern	n	%	Pattern	n	%
11111	48	18.0	21221	5	1.9	11312	3	1.1
11211	8	3.0	31311	5	1.9	11322	3	1.1
11222	8	3.0	33311	5	1.9	12111	3	1.1
11121	7	2.6	11311	4	1.5	12211	3	1.1
11131	7	2.6	33333	4	1.5	21212	3	1.1
21211	7	2.6	33433	4	1.5	31131	3	1.1
11112	6	2.3	11113	3	1.1	32231	3	1.1
21222	6	2.3	11212	3	1.1	32331	3	1.1
11122	5	1.9	11221	3	1.1	33331	3	1.1

[1]Given by more ≥3 patients.
[2]Only questionnaires with responses in all five dimensions were used for this analysis (266 (98%) of 272).
[3]Defined as EQ-5D questionnaires filled out at the start of cycle 1 or 2.

Supplemental Table S14. Most frequent response patterns[1] of all EQ-5D questionnaires.[2]

Pattern	n	%	Pattern	n	%	Pattern	n	%
11111	414	29.5	33533	8	0.6	31111	4	0.3
11121	82	5.8	22231	7	0.5	31211	4	0.3
21221	35	2.5	22232	7	0.5	31232	4	0.3
11112	31	2.2	22322	7	0.5	32322	4	0.3
11211	30	2.1	33311	7	0.5	32331	4	0.3
11221	24	1.7	11114	6	0.4	33432	4	0.3
11131	23	1.6	11311	6	0.4	33433	4	0.3
22221	21	1.5	21122	6	0.4	43443	4	0.3
11122	19	1.4	22321	6	0.4	11322	3	0.2
21211	19	1.4	22332	6	0.4	11522	3	0.2
11113	18	1.3	44432	6	0.4	21123	3	0.2
11212	17	1.2	44433	6	0.4	21233	3	0.2
33333	17	1.2	11231	5	0.4	22331	3	0.2
11222	15	1.1	11313	5	0.4	31113	3	0.2
21111	15	1.1	12211	5	0.4	31221	3	0.2
33331	14	1.0	21223	5	0.4	31332	3	0.2
21121	13	0.9	21231	5	0.4	32231	3	0.2
21222	13	0.9	22323	5	0.4	32232	3	0.2
31311	11	0.8	22333	5	0.4	32321	3	0.2
31331	11	0.8	32332	5	0.4	33321	3	0.2
21212	10	0.7	43433	5	0.4	33342	3	0.2
22222	9	0.6	11312	4	0.3	33442	3	0.2
31131	9	0.6	11333	4	0.3	33553	3	0.2
33332	9	0.6	21232	4	0.3	35553	3	0.2
11232	8	0.6	21311	4	0.3	41111	3	0.2
11331	8	0.6	22211	4	0.3	42433	3	0.2
31333	8	0.6	22223	4	0.3	44543	3	0.2

[1]Given by more ≥3 patients.
[2]Only questionnaires with responses in all five dimensions were used for this analysis (1402 (97%) of 1450).

Supplemental Table S15. Overview of EQ-5D responses[1] by patient group and responder status.

	Patients with EQ-5D during azacitidine (n=272)	Patients with EQ-5D at azacitidine start (n=205)
Mean no. of EQ-5D per patient, n (SD)	5.4 (6.2)	4.9 (6.2)
Median (IQR)	3.0 (1.0-7.0)	3.0 (1.0-5.0)
Min-max	1.0-44.0	1.0-44.0
Unknown, n (%)	0 (0.0)	0 (0.0)
Included patients, n (%)	272 (100.0)	205 (100.0)
Responders: Mean no. of EQ-5D per patient, n (SD)	7.0 (7.1)	7.2 (7.8)
Median (IQR)	4.0 (2.0-10.0)	4.0 (2.0-9.5)
Min-max	1.0-44.0	1.0-44.0
Unknown, n (%)	0 (0.0)	0 (0.0)
Included patients, n (%)	161 (100.0)	104 (100.0)
Non-responders: Mean no. of EQ-5D per patient, n (SD)	3.0 (3.5)	2.6 (2.5)
Median (IQR)	2.0 (1.0-4.0)	2.0 (1.0-3.0)
Min-max	1.0-25.0	1.0-13.0
Unknown, n (%)	0 (0.0)	0 (0.0)
Included patients, n (%)	111 (100.0)	101 (100.0)
No. of patients with EQ-5D in: Cycles 1-2, n/n (%)	205/272 (75.4)	205/205 (100.0)
Cycles 3-6	147/272 (54.0)	113/205 (55.1)
Cycles 7-12	110/272 (40.4)	70/205 (34.1)
Cycles ≥13	78/272 (28.7)	40/205 (19.5)
Unknown	0/272 (0.0)	0/205 (0.0)
Responders: No. of patients with EQ-5D in: Cycles 1-2, n/n (%)	104/161 (64.6)	104/104 (100.0)
Cycles 3-6	101/161 (62.7)	75/104 (72.1)
Cycles 7-12	86/161 (53.4)	52/104 (50.0)
Cycles ≥13	68/161 (42.2)	34/104 (32.7)
Unknown	0/161 (0.0)	0/104 (0.0)
Non-responders: No. of patients with EQ-5D in: Cycles 1-2, n/n (%)	101/111 (91.0)	101/101(100.0)
Cycles 3-6	46/111 (41.4)	38/101 (37.6)
Cycles 7-12	24/111 (21.6)	18/101 (17.8)
Cycles ≥13	10/111 (9.0)	6/101 (5.9)
Unknown	0/111 (0.0)	0/101 (0.0)

SD indicates standard deviation; IQR, interquartile range.

[1]225 (15%) of 1456 EQ-5D-3l questionnaires were collected during January 2012 to June 2013; thereafter 1231 (85%) of EQ-5D-5L questionnaires were collected.

Supplemental Table S16. Prevalence of problems as assessed by EQ-5D[1] at azacitidine treatment start (n=205).

	Mobility problem[2]		Selfcare problem[2]		Usual activities problem[2]		Pain/discomfort problem[2]		Anxiety/depression problem[2]		Level Sum Score[3]			Index value[4]			EQ-VAS		
	n/n (%)	p[5]	n/n (%)	p[5]	n/n (%)	p[5]	n/n (%)	p[5]	n/n (%)	p[5]	n	Mean (SD)	p[5]	n	Mean (SD)	p[6]	n	Mean (SD)	p[6]
**Total cohort**																			
1st available EQ-5D	136/272 (50.0)	NA	68/72 (25.0)	NA	150/272 (55.1)	NA	138/272 (50.7)	NA	125/272 (46.0)	NA	266	9.1 (3.9)	NA	266	0.807 (0.232)	NA	263	63.9 (21.6)	NA
EQ-5D in cycle 1 or 2	104/205 (50.7)	NA	46/205 (22.4)	NA	120/205 (58.5)	NA	102/205 (49.8)	NA	100/205 (48.8)	NA	200	9.2 (3.9)	NA	198	0.810 (0.229)	NA	200	64.5 (21.4)	NA
**Disease-related parameters**																			
Azacitidine ≥2nd line: No<br>Yes	75/145 (52.1)<br>29/59 (49.2)	0.7045	35/143 (24.5)<br>11/59 (18.6)	0.3688	86/141 (61.0)<br>34/59 (557.6)	0.6577	76/143 (53.1)<br>26/59 (44.1)	0.2406	72/143 (50.3)<br>28/59 (47.5)	0.7085	141<br>59	9.3 (4.0)<br>8.7 (3.5)	0.3288	141<br>59	0.800 (0.243)<br>0.831 (0.192)	0.4282	141<br>57	63.3 (22.0)<br>67.5 (19.7)	0.2136
Diagnosis: MDS or CMML<br>AML	59/112 (52.7)<br>45/91 (49.5)	0.6472	28/111 (25.2)<br>18/91 (19.8)	0.3585	66/109 (60.6)<br>54/91 (59.3)	0.8619	66/111 (59.5)<br>36/91 (39.6)	0.0049	53/111 (47.4)<br>47/91 (51.6)	0.5812	109<br>91	9.4 (4.0)<br>8.8 (3.6)	0.2921	109<br>91	0.788 (0.256)<br>0.835 (0.192)	0.2160	110<br>88	64.4 (21.2)<br>64.7 (21.7)	0.9440
Treatment-related disease: No<br>Yes	89/175 (50.9)<br>12/24 (50.0)	0.9372	39/174 (22.4)<br>6/24 (25.0)	0.7769	102/172 (59.3)<br>14/24 (58.3)	0.9279	84/174 (48.3)<br>15/24 (62.5)	0.1914	79/174 (45.4)<br>17/24 (70.8)	0.0194	172<br>24	9.1 (3.9)<br>9.5 (3.7)	0.4741	172<br>24	0.810 (0.238)<br>0.809 (0.182)	0.4869	170<br>24	64.7 (21.8)<br>64.4 (19.9)	0.7998
IPSS: Low or intermediate-1<br>Intermediate-2 or high	39/72 (54.2)<br>62/125 (49.6)	0.5369	17/71 (23.9)<br>27/125 (21.6)	0.7055	40/69 (58.0)<br>75/125 (60.0)	0.7830	39/71 (54.9)<br>58/125 (46.4)	0.2510	32/71 (45.1)<br>64/125 (51.2)	0.4093	69<br>125	9.3 (4.2)<br>8.9 (3.5)	0.7663	69<br>125	0.789 (0.274)<br>0.836 (0.169)	0.7950	70<br>122	65.6 (20.7)<br>64.6 (21.8)	0.6783
R-IPSS: Very low or low<br>Intermediate, poor, very poor	11/26 (42.3)<br>90/169 (53.3)	0.2984	6/26 (23.1)<br>39/168 (23.2)	0.9877	13/26 (50.0)<br>102/166 (61.4)	0.2682	15/26 (57.7)<br>82/168 (48.8)	0.3992	12/26 (46.2)<br>84/168 (50.0)	0.7151	26<br>166	9.3 5(0.)<br>9.1 (3.6)	0.5927	26<br>166	0.758 (0.369)<br>0.821 (0.188)	0.7551	25<br>165	64.6 (21.8)<br>64.7 (21.1)	0.9609
IPSS cytogenetic risk: good<br>Intermediate or poor	60/125 (48.0)<br>34/56 (60.7)	0.1135	29/124 (23.4)<br>14/56 (25.0)	0.8143	67/123 (54.5)<br>35/55 (63.6)	0.2534	62/124 (50.0)<br>29/56 (51.8)	0.8244	64/124 (50.0)<br>27/56 (48.2)	0.6729	123<br>55	9.0 (3.9)<br>9.5 (3.8)	0.2706	123<br>55	0.814 (0.228)<br>0.806 (0.216)	0.3255	122<br>54	65.7 (21.4)<br>64.1 (21.1)	0.6006
Peripheral blood blasts <10%<br>≥10%	78/156 (50.0)<br>26/47 (55.3)	0.5225	34/155 (21.9)<br>12/47 (25.5)	0.6065	94/153 (61.4)<br>26/47 (55.3)	0.4539	83/155 (53.5)<br>19/47 (40.4)	0.1150	77/155 (49.7)<br>23/47 (48.9)	0.9291	153<br>47	9.3 (4.0)<br>8.8 (3.3)	0.7245	153<br>47	0.798 (0.246)<br>0.847 (0.162)	0.4270	153<br>45	64.8 (20.9)<br>63.8 (23.1)	0.7879
Monocytes <10%<br>≥10%	56/121 (46.3)<br>44/75 (58.7)	0.0918	23/121 (19.0)<br>22/74 (29.7)	0.0846	61/119 (51.3)<br>52/74 (70.3)	0.0091	60/121 (49.6)<br>41/74 (55.4)	0.4301	52/121 (43.0)<br>43/74 (58.1)	0.0402	119<br>74	8.5 (3.5)<br>10.1 (4.3)	0.0053	119<br>74	0.850 (0.193)<br>0.752 (0.267)	0.0052	118<br>73	67.7 (19.8)<br>61.5 (22.5)	0.0626
Haemoglobin <10.0 g/dL<br>≥10.0 g/dL	81/142 (57.0)<br>23/61 (37.7)	0.0115	37/141 (26.2)<br>9/61 (14.8)	0.0739	89/139 (64.0)<br>31/61 (50.8)	0.0792	73/141 (51.8)<br>29/61 (47.5)	0.5807	71/141 (50.4)<br>29/61 (47.5)	0.7135	139<br>61	9.5 (4.0)<br>8.3 (3.5)	0.0295	139<br>61	0.790 (0.242)<br>0.855 (0.191)	0.0429	137<br>61	62.8 (21.0)<br>68.5 (21.8)	0.0545
Red blood cell transfusions ≤3<br>>3	62/138 (44.9)<br>22/26 (84.6)	0.0002	31/137 (22.6)<br>8/26 (30.8)	0.3724	75/135 (55.6)<br>20/26 (76.9)	0.0425	73/137 (53.3)<br>16/26 (61.5)	0.4384	64/137 (46.7)<br>15/26 (57.7)	0.3045	135<br>26	9.0 (4.0)<br>9.9 (3.2)	0.0723	135<br>26	0.809 (0.247)<br>0.864 (0.163)	0.1412	134<br>25	65.6 (21.7)<br>55.2 (17.6)	0.0147
Platelet count <100 G/L<br>≥100 G/L	36/65 (55.4)<br>68/138 (49.3)	0.4165	17/65 (26.2)<br>29/137 (21.2)	0.4299	39/63 (61.9)<br>81/137 (59.1)	0.7092	34/65 (52.3)<br>68/137 (49.6)	0.7226	30/65 (46.2)<br>70/137 (51.1)	0.5117	63<br>137	9.4 (4.0)<br>9.0 (3.8)	0.4665	61<br>137	0.797 (0.254)<br>0.815 (0.218)	0.4980	64<br>134	65.8 (19.9)<br>63.9 (22.1)	0.6100
**Patient-related parameters**																			
Sex male: No<br>Yes	45/81 (55.6)<br>59/122 (48.4)	0.3152	21/81 (25.9)<br>25/121 (20.7)	0.3819	49/79 (62.0)<br>71/121 (58.7)	0.6366	44/81 (54.3)<br>58/121 (47.9)	0.3735	47/81 (58.0)<br>53/121 (43.8)	0.0475	79<br>121	9.6 (4.0)<br>8.9 (3.7)	0.1644	79<br>121	0.786 (0.261)<br>0.825 (0.206)	0.2445	77<br>121	66.3 (21.9)<br>63.4 (21.1)	0.2408
Age ≥75 yrs: No<br>Yes	47/105 (44.8)<br>57/98 (58.2)	0.0563	19/104 (18.3)<br>27/89 (27.6)	0.1159	64/103 (62.1)<br>56/97 (57.7)	0.5252	44/104 (42.3)<br>59/98 (59.2)	0.0165	51/104 (49.0)<br>49/98 (50.0)	0.8913	103<br>97	8.7 (3.4)<br>9.6 (4.2)	0.2478	103<br>97	0.832 (0.191)<br>0.785 (0.263)	0.2429	103<br>95	66.9 (21.0)<br>60.0 (21.6)	0.1083
ECOG-PS: 0-1<br>≥2	74/163 (45.4)<br>30/40 (75.0)	0.0008	26/162 (16.0)<br>20/40 (50.0)	<.0001	87/160 (54.4)<br>33/40 (82.5)	0.0012	79/162 (48.8)<br>23/40 (57.5)	0.3224	70/162 (43.2)<br>30/40 (75.0)	0.0003	160<br>40	8.4 (3.4)<br>12.0 (4.3)	<.0001	160<br>40	0.847 (0.185)<br>0.659 (0.315)	<.0001	159<br>39	66.5 (20.8)<br>56.6 (22.3)	0.0092
HCT-CI: Low risk<br>Intermediate risk<br>High risk	31/77 (40.3)<br>33/65 (50.8)<br>40/61 (65.6)	0.0127	13/77 (16.9)<br>12/65 (18.5)<br>21/60 (35.0)	0.0259	40/75 (53.3)<br>36/65 (55.4)<br>44/60 (73.3)	0.0406	38/77 (49.4)<br>26/65 (40.0)<br>38/60 (63.3)	0.0324	36/77 (46.8)<br>30/65 (46.2)<br>34/60 (56.7)	0.4155	75<br>65<br>60	8.3 (3.3)<br>8.9 (3.7)<br>10.4 (4.4)	0.0133	75<br>65<br>60	0.849 (0.186)<br>0.822 (0.224)<br>0.748 (0.271)	0.0189	75<br>64<br>59	67.8 (20.1)<br>65.4 (21.0)<br>59.5 (22.7)	0.0750
No. of comorbidities: 0-1<br>≥2	52/116 (44.8)<br>52/87 (59.8)	0.0350	23/116 (19.8)<br>23/86 (26.7)	0.2464	66/114 (57.9)<br>54/86 (62.8)	0.4841	57/116 (49.1)<br>45/86 (52.3)	0.6541	52/116 (44.8)<br>48/86 (55.8)	0.1225	114<br>86	8.7 (3.5)<br>9.8 (4.3)	0.0689	114<br>86	0.839 (0.183)<br>0.770 (0.276)	0.0703	113<br>85	66.8 (21.0)<br>61.6 (21.6)	0.0829

IPSS indicates International Prognostic Scoring System ; IPSS-LR, IPSS lower-risk; IPSS-HR, IPSS higher-risk; R-IPSS, revised IPSS; ECOG-PS indicates Eastern Cooperative Oncology Group Performance Score; HCT-CI, Haematopoietic Stem Cell Comorbidity Index; MRC, Medical research Council.

[1]First available EQ-5D.
[2]Problems were defined as answer options 2, 3, 4, or 5 for EQ-5D-5L and answer options 2 or 3 for EQ-5D-3L.
[3]Represents the numerical sum of all EQ-5D responses.
[4]The EQ-5D-5L index is measured on a scale from 0 to 1, whereby 0 indicates death and 1 perfect health.
[5]Baseline parameters and EQ-5D-5L results were compared using the Chi-squared test (based on non-missing observations) for EQ-5D-5L problems (=2,3,4,5) vs EQ-5D-5L no-problems (=1).
[6]Baseline parameters and EQ-5D-5L results were compared using the Wilcoxon rank-sum test (also called Mann-Whitney-U test or Mann-Whitney-Wilcoxon Test) for Level Sum Score, EQ-5D-5L index value and EQ-VAS.

Supplemental Table S17. Multivariate-adjusted[1] longitudinal analyses of EQ-5D results and dichotomised parameters per azacitidine treatment cycle using mixed-effects linear models.

	Mobility		Selfcare		Usual activities		Pain/discomfort		Anxiety/depression		Level Sum Score[2]		EQ-VAS		EQ-5D-5L Index	
**Differential blood count**	n[3]	p[4]	n	p	n	p	n	p	n	p	n	p	n	p	n	p
Peripheral blood blasts < vs ≥ 5%	1425	0.9897	1417	0.2548	1417	0.1447	1421	0.9703	1416	0.8775	1395	0.2930	1365	0.0996	1395	0.3916
White blood cell count < vs ≥ 30.0 G/L	1429	0.1502	1421	0.5278	1421	0.2869	1425	0.0801	1420	0.2674	1399	0.1371	1368	0.7712	1399	0.1272
Absolute neutrophil count < vs ≥ 1.0 G/L	1415	0.2206	1407	0.1586	1407	0.8529	1411	0.6784	1406	0.6362	1385	0.5171	1355	0.1329	1385	0.9389
Monocytes < vs ≥ 1.0 G/L	1417	0.2559	1409	0.9738	1409	0.4770	1413	0.5203	1408	0.8287	1387	0.6366	1357	0.2476	1387	0.9439
Lymphocytes < vs ≥ 1.0 G/L	1402	0.4021	1394	0.5043	1394	0.6879	1398	0.5349	1393	0.0941	1372	0.8871	1343	0.5429	1372	0.6557
Haemoglobin < vs ≥ 10.0 g/dL	1429	<0.0001	1421	0.0227	1421	<0.0001	1425	0.9289	1420	0.7871	1399	<0.0001	1368	<0.0001	1399	0.0110
Red blood cell transfusions: Yes vs No	1429	0.0003	1421	0.7072	1421	<0.0001	1425	0.1935	1420	0.6996	1399	0.0003	1368	<0.0001	1399	0.0161
Platelet count < vs ≥ 50 G/L	1429	0.0122	1421	0.0647	1421	0.0248	1425	0.3142	1420	0.9574	1399	0.0212	1368	0.0006	1399	0.0156
Platelet transfusions: Yes vs No	1429	0.0257	1421	0.0047	1421	0.0044	1425	0.0002	1420	0.2067	1399	0.0002	1368	<0.0001	1399	<0.0001
**Comorbidity/toxicity**																
Ferritin < vs ≥ 1000 µg/L	723	0.0006	720	0.0598	720	0.0020	722	0.0785	718	0.5635	709	0.0024	703	0.0053	709	0.0163
Creatinine < vs ≥ 1.5 mg/dL	1417	0.7976	1409	0.8133	1409	0.6386	1413	0.7286	1408	0.7550	1387	0.9162	1356	0.5338	1387	0.8874
Lactate dehydrogenase, U/L	1399	0.4066	1391	0.1095	1392	0.7977	1395	0.0642	1390	0.9778	1370	0.3834	1337	0.3343	1370	0.3673
Glutamate oxaloacetate transaminase, U/L	1406	0.7039	1398	0.8181	1399	0.5276	1402	0.2078	1397	0.4316	1377	0.6822	1345	0.5734	1377	0.9119
Glutamate pyruvate transaminase, U/L	1348	0.0867	1340	0.9662	1340	0.6501	1344	0.4822	1339	0.8201	1318	0.4770	1288	0.7212	1318	0.7369
Bilirubin < vs ≥ 1.2 mg/dL	1407	0.0149	1399	0.0066	1399	0.0451	1403	0.9600	1398	0.4338	1377	0.0158	1346	0.0494	1377	0.0170
Albumin < vs ≥ 3.4 mg/dL	583	0.0052	579	<0.0001	578	0.0412	580	0.0942	576	0.0454	567	0.0034	565	0.2309	567	0.0355
Cholinesterase < vs ≥ 3.7 U/L	584	0.0108	581	0.0437	580	0.6728	582	0.1706	580	0.5751	567	0.0992	567	0.0216	567	0.7691
Adverse events[4] Grade 0-2 vs 3-4	1429	0.0208	1421	0.0616	1421	0.0229	1425	0.0028	1420	0.0179	1399	0.0005	1368	0.0074	1399	<0.0001
**Azacitidine dose/regimen**																
Azacitidine < vs ≥ 7 days	1429	0.1648	1421	0.0129	1421	0.4369	1425	0.0964	1420	0.0158	1399	0.0096	1368	0.4788	1399	0.0288
Azacitidine < vs ≥ 75 mg/m²/day	1426	0.1485	1418	0.1155	1418	0.0249	1422	0.0168	1417	0.0001	1396	0.0003	1365	0.0040	1396	0.0013
**Heamatologic improvement (HI)**																
HI-any[5]: Yes vs No	1275	0.0004	1268	0.0130	1270	0.0003	1272	0.6473	1266	0.1747	1248	0.0005	1221	<0.0001	1248	0.0048
HI-Erythrocytes: Yes vs No	1296	0.0008	1289	0.0163	1291	<0.0001	1293	0.2981	1287	0.7419	1269	0.0084	1239	<0.0001	1269	0.1645
HI-Platelets: Yes vs No	1317	0.0025	1310	0.0011	1311	0.0008	1315	0.0951	1310	0.2232	1288	0.0005	1262	<0.0001	1288	0.0003
HI-Neutrophils: Yes vs No	1362	0.4299	1355	0.7016	1354	0.2083	1358	0.1326	1353	0.4239	1333	0.2837	1303	0.0012	1333	0.6162

[1]Adjusted for the covariates remaining in the final Cox model: ECOG-PS, number of comorbidities, platelet count/transfusion dependence, peripheral blood blasts, azacitidine treatment line, and azacitidine dose in cycle one. [2]Represents the numerical sum of all EQ-5D-5L responses.
[3]Number of parameter/EQ-5D-5L response pairs. [4]Assessed according to CTCAEv4.0. [5]Includes HI-Neutrophils and/or HI-Erythrocytes and/or HI-Platelets.

Supplemental Table S18. Multivariate-adjusted[1] longitudinal analyses of EQ-5D-5L results and continuous parameters per azacitidine treatment cycle using mixed-effects linear models.

	Mobility		Selfcare		Usual activities		Pain/discomfort		Anxiety/depression		Level Sum Score[2]		EQ-VAS		EQ-5D-5L Index	
**Differential blood count**	n[3]	p[4]	n	p	n	p	n	p	n	p	n	p	n	p	n	p
Peripheral blood blasts, %	1425	0.2931	1417	0.1173	1417	0.6567	1421	0.2455	1416	0.8849	1395	0.1666	1365	0.3326	1395	0.3276
White blood cell count, G/L	1429	0.0510	1421	0.3972	1421	0.7540	1425	0.1823	1420	0.5275	1399	0.4196	1368	0.7016	1399	0.5760
Absolute neutrophil count, G/L	1415	0.3370	1407	0.5488	1407	0.3411	1411	0.4599	1406	0.0845	1385	0.6824	1355	0.9833	1385	0.7849
Monocytes, G/L	1417	0.2702	1409	0.9889	1409	0.9073	1413	0.3187	1408	0.8991	1387	0.8092	1357	0.3015	1387	0.6981
Lymphocytes, G/L	1402	0.0953	1394	0.4325	1394	0.2533	1398	0.1598	1393	0.8844	1372	0.1766	1343	0.9788	1372	0.2789
Haemoglobin, g/dL	1429	<0.0001	1421	0.2380	1421	<0.0001	1425	0.6100	1420	0.8262	1399	0.0004	1368	<0.0001	1399	0.0281
Red blood cell transfusions, n	1429	0.0010	1421	0.4481	1421	<0.0001	1425	0.0337	1420	0.2105	1399	<0.0001	1368	<0.0001	1399	<0.0001
Platelet count, G/L	1429	0.0384	1421	0.2697	1421	0.0182	1425	0.8890	1420	0.1057	1399	0.0457	1368	0.0113	1399	0.0485
Platelet transfusions, n	1429	0.4779	1421	0.3081	1421	0.0106	1425	0.0472	1420	0.1188	1399	0.0190	1368	<0.0001	1399	0.0098
**Comorbidity/toxicity**																
Ferritin, µg/L	723	0.0381	720	0.5788	720	0.0546	722	0.0887	718	0.6147	709	0.0995	703	0.0014	709	0.3487
Creatinine, mg/dL	1417	0.2134	1409	0.6361	1409	0.3296	1413	0.1361	1408	0.6411	1387	0.1302	1356	0.3480	1387	0.1784
Lactate dehydrogenase, U/L	1399	0.1668	1391	0.0649	1392	0.8868	1395	0.6800	1390	0.8102	1370	0.5183	1337	0.2881	1370	0.6999
Glutamate oxaloacetate transaminase, U/L	1406	0.5974	1398	0.6828	1399	0.5273	1402	0.9873	1397	0.2166	1377	0.5676	1345	0.7348	1377	0.7793
Glutamate pyruvate transaminase, U/L	1348	0.2978	1340	0.4089	1340	0.6598	1344	0.7476	1339	0.1711	1318	0.6358	1288	0.3041	1318	0.9834
Bilirubin, mg/dL	1407	0.0185	1399	0.3843	1399	0.0699	1403	0.2891	1398	0.3214	1377	0.1404	1346	0.2159	1377	0.0756
Albumin, mg/dL	583	0.0018	579	0.0035	578	0.0112	580	0.0701	576	0.0087	567	0.0014	565	0.0011	567	0.0030
Cholinesterase, U/L	584	0.0070	581	0.0335	580	0.0063	582	0.7173	580	0.5278	567	0.0127	567	0.0014	567	0.0157
Adverse events[4], grade 0-4	1429	0.0125	1421	0.0977	1421	0.0114	1425	0.0041	1420	0.1800	1399	0.0023	1368	0.0006	1399	0.0008
**Azacitidine dose/regimen**																
Azacitidine, number of days	1429	0.1297	1421	0.0072	1421	0.5377	1425	0.0660	1420	0.0037	1399	0.0044	1368	0.9211	1399	0.0056
Azacitidine dose, $mg/m^2/day$	1426	0.9196	1418	0.0426	1418	0.0723	1422	0.1298	1417	0.0246	1396	0.0081	1365	0.0848	1396	0.0275

EQ-VAS indicates EuroQol Visual Analogue Scale.

[1]Adjusted for the covariates remaining in the final Cox model: ECOG-PS, number of comorbidities, platelet count/transfusion dependence, peripheral blood blasts, azacitidine treatment line, and azacitidine dose in cycle one.
[2]Represents the numerical sum of all EQ-5D responses.
[3]Number of parameter/EQ-5D response pairs.
[4]Assessed according to CTCAEv4.0.

Supplemental Figure S1. Heatmap of p-values resulting from multivariate-adjusted mixed-effect linear models of longitudinally assessed EQ-5D-5L responses and concomitantly assessed continuous clinical parameters.

	Mobility	Selfcare	Usual activities	Pain/discomfort	Anxiety/depression	Level Sum Score	EQ-VAS	EQ-5D-5L Index
Peripheral blood blasts, %	0.29	0.12	0.66	0.25	0.88	0.17	0.33	0.33
White blood cell count, G/L	0.051	0.4	0.75	0.18	0.53	0.42	0.7	0.58
Absolute neutrophil count, G/L	0.34	0.55	0.34	0.46	0.084	0.68	0.98	0.78
Monocytes, G/L	0.27	0.99	0.91	0.32	0.9	0.81	0.3	0.7
Lymphocytes, G/L	0.065	7.5e-05	0.00094	0.2	0.067	0.00026	0.092	0.00022
Haemoglobin, g/dL	9e-07	0.24	3.3e-06	0.61	0.83	0.00036	8.9e-13	0.028
Red blood cell transfusions, n	0.001	0.45	7.3e-09	0.034	0.21	2.2e-05	9.1e-14	8.3e-05
Platelet count, G/L	0.038	0.27	0.018	0.89	0.11	0.046	0.011	0.048
Platelet transfusions, n	0.48	0.31	0.011	0.047	0.12	0.019	2.3e-06	0.0098
Ferritin, µg/L	0.038	0.58	0.055	0.089	0.61	0.1	0.0014	0.35
Creatinine, mg/dL	0.21	0.64	0.33	0.14	0.64	0.13	0.35	0.18
Lactate dehydrogenase, U/L	0.17	0.065	0.89	0.68	0.81	0.52	0.29	0.7
Glutamate oxaloacetate transaminase, U/L	0.6	0.68	0.53	0.99	0.22	0.57	0.73	0.78
Glutamate pyruvate transaminase, U/L	0.3	0.41	0.66	0.75	0.17	0.64	0.3	0.98
Bilirubin, mg/dL	0.018	0.38	0.07	0.29	0.32	0.14	0.22	0.076
Albumin, mg/dL	0.0018	0.0035	0.011	0.07	0.0087	0.0014	0.0011	0.003
Cholinesterase, U/L	0.007	0.034	0.0063	0.72	0.53	0.013	0.0014	0.016
Adverse events, grade 0-4	0.013	0.098	0.011	0.0041	0.18	0.0023	0.00061	0.0008
Azacitidine, number of days	0.13	0.0072	0.54	0.066	0.0037	0.0044	0.92	0.0056
Azacitidine dose, mg/m2/day	0.97	0.012	0.021	0.11	0.017	0.0037	0.23	0.0092

The individual boxes contain the p-values of the corresponding multivariate-adjusted mixed effect linear models using EQ-5D-5L responses as endogenous variables (x-axis), and various clinical measurements as exogenous variables (y-axis). Multivariate adjustment was performed by admitting the following variables remaining in the final Cox model as covariates: ECOG-PS, number of comorbidities, platelet count ≤30 G/L or platelet transfusion dependence, peripheral blood blasts, azacitidine treatment line, and azacitidine dose in cycle one.

LAND SALZBURG

Frau
Dr. Barbara Meininger

Ethikkommission

Zahl (Bitte im Antwortschreiben anführen)
415-EP/39/30-2016
Betreff
Bestätigung

Datum
16.03.2016

Sebastian-Stief-Gasse 2
Postfach 527 | 5010 Salzburg
Fax +43 662 8042-2929
ethikkommission@salzburg.gv.at
Mag. Silvia Peterbauer
Telefon

Sehr geehrte Frau Dr. Meininger!

Die ständigen Mitglieder der Ethikkommission für das Bundesland Salzburg haben

- EK Meldung unterzeichnet am 05.01.2016
- Protokoll Version 4 vom 11.09.2015
  Zusatz vom 16.03.2016 zum Protokoll V.4
- Sponsor Approval Page unterzeichnet am 13.01.2016
- Protokoll Version 4 vom 11.09.2015 tracking version
- Patienteninformation Version 4.0 vom 04.11.2015 (Änderung gelb markiert)
- Patienteninformation Genetik Version 5.1 vom 16.03.2016
- Patienteninformation Genetik Version 5.1 vom 16.03.2016 Tracking Version

zum klinischen Forschungsprojekt mit dem Titel "Registry on Hympomethylating Agents in Myeloid Neoplasms, including Myelodysplastic Syndrome (MDS), CMML and AML", zustimmend zur Kenntnis genommen.

Mit besten Grüßen
Für die Ethikkommission
Mag. Ulrike Wendl-Toiflhart
Amtssigniert. Hinweise zur Prüfung der Amtssignatur finden Sie unter www.salzburg.gv.at/amtssignatur

www.salzburg.gv.at

Amt der Salzburger Landesregierung | Ethikkommission
Postfach 527 | 5010 Salzburg | Österreich | Telefon +43 662 8042 0* | post@salzburg.gv.at | DVR 0078182

PATIENT REGISTRY

“Registry on Hypomethylating Agents in Myeloid Neoplasms, including Myelodysplastic Syndromes (MDS), Chronic Myelominocytic Leukemia (CMML) and Acute Myeloid Leukemia (AML)“

Prim. Univ. Prof. Dr. R. Greil / PD Dr. DI Lisa Pleyer

Registry Version: 4
Registry Date: 11.09.2015

sponsored by

AGMT | ARBEITSGEMEINSCHAFT MEDIKAMENTÖSE TUMORTHERAPIE

COORDINATING INVESTIGATOR
Prim. Univ. Prof. Dr. Richard Greil
Universitätsklinik für Innere Medizin III
Universitätsklinikum der PMU
Salzburger Landeskliniken
Müllner Hauptstraße 48, 5020 Salzburg
Tel.: 0662/4482-2879
e-mail: r.greil@salk.at

PROTOCOL CONTACT PERSON:
Priv.Doz. Dr. med., DI biomed. Inf. Lisa Pleyer
Universitätsklinik für Innere Medizin III
Universitätsklinikum der PMU
Salzburger Landeskliniken
Müllner Hauptstraße 48, 5020 Salzburg
Tel.: 0662/4482-58271
e-mail: l.pleyer@salk.at

SPONSOR:
Arbeitsgemeinschaft medikamentöse Tumortherapie gemeinnützige GmbH
Klinisch-wissenschaftlicher Geschäftsführer: Prim. Univ. Prof. Dr. Richard Greil
Plenergasse 5/31
1180 Wien
Tel.: 0662/4482-2879
e-mail: r.greil@salk.at

MANAGEMENT:
Arbeitsgemeinschaft medikamentöse Tumortherapie gemeinnützige GmbH
Dr. Daniela Wolkersdorfer
Plenergasse 5/31
1180 Wien
Tel.: 0664/1425504
Fax.: 0662/482-4174
e-mail: d.wolkersdorfer@agmt.at

SPONSOR APPROVAL PAGE

13. Jan. 2016

Prof. Dr. Richard Greil — Date

Klinisch-wissenschaftlicher Geschäftsführer der AGMT and Coordinating Investigator

13. Jan. 2016

Priv. Doz. Dr. DI. Lisa Pleyer — Date

Co-Investigator and Protocol Contact

TABLE OF CONTENT

1. BACKGROUND AND RATIONALE ... *5*

2. STUDY DESIGN ... *7*

3. OBJECTIVES ... *8*

4. INCLUSION AND EXCLUSION CRITERIA ... *8*

5. ASSESSSMENTS ... *8*

6. ADVERSE EVENTS ... *9*

7. STATISTICAL ANALYSES ... *11*

8. INFORMED CONSENT ... *11*

1. BACKGROUND AND RATIONALE

Myelodysplastic syndromes (MDS) are a group of haematologic malignancies that affect approximately 300,000 people worldwide. In the French American British (FAB) Classification, chronic myelomonocytic leukemia (CMML) was considered to be a subtype of MDS. In 2001 CMML was considered to be a distinct disease entity by the World Health organization (WHO) Classification. The incidence and prevalence of MDS has been rising in the last years, due to earlier diagnosis, heightened awareness of many physicians to changes in the differential blood count of elderly people, as well as due to the increasing age of the population. MDS comprises a group of heterogenous diseases, commonly characterized by dysplastic hematopietic cells in the bone marrow as well as peripheral blood. Dysplastic hematopoietic progenitor cells in the bone marrow are incapable of fully differentiating, and are characterized by an enhanced apoptosis rate. This results in inefficient hematopoiesis. Early stage MDS is characterized by hyperplastic bone marrow, which is in stark contrast to the various cytopenias observed in the peripheral blood. Approximately one third of all MDS cases eventually progresses to full-blown acute myeloid leukemia (AML). Cytopenias may affect one or more cell lines, resulting in predominating thrombocytopenia, anemia and/or leukopenia, which is correlated with an enhanced risk of bleeding, tissue hypoxia and an enhanced rate of potentially life threatening infectious complications. MDS patients become dependent on erythrocyte and/or platelet transfusions at some stage during the couse of their disease. As a consequence of frequent transfusions, iron overload and/or generation of alloantibodies, which complicate the search for matching blood products, are common complications. MDS classification is based on the WHO classification (Table 1) and risk stratification currently relies on the International Prognostic Scoring Index (IPSS) (Table 2). Patients with MDS have a median overall survival rate of 2-3 years, and patients with higher-risk MDS have a median survival of approximately 6-12 months underscoring the imminent need for efficacious therapies and for longterm monitoring of response rates as well as toxicity.

In 2008 the European Medicines Agency (EMA) granted VIDAZA® (azacitidine) full marketing authorization for the treatment of patients with higher-risk MDS and AML with 20-30% bone marrow blasts. The approval was based upon efficacy and safety data from the MDS-001 trial, the largest, international randomized Phase III controlled study ever conducted in higher-risk MDS and low bone marrow blast count AML patients, demonstrating a clinically relevant increase in median survival of 9.4 months (24.4 vs. 15 months) as compared to conventional best supportive care regimens. In addition to extending overall survival, 45 percent of azacitidine treated patients achieved red blood cell transfusion independence in the AZA-001 study. Azacitidine was well-tolerated by patients.

In October 2015 EMA expanded approval of azacitidine to include AML patients with >30% bone marrow blasts.

The Food and Drug Agency (FDA) granted approval DACOGEN® (decitabine) approval for the treatment of MDS, but not for AML. In contrast, EMA granted DACOGEN® (decitabine) approval for the treatment of AML, irrespective of bone marrow blast count in 2012, but approval for MDS was not granted. Approval by EMA for AML was based on a post-hoc analysis of a multicenter, randomized, open-label phase III study (DACO-016) in patients ≥ 65 years with newly diagnosed AML, which showed a slight survival advantage for decitabine over conventional care arms.

Table 1: WHO-Classification of the Myelodysplastic Syndromes

Type of MDS	Peripheral blood anomalies	Bone marrow anomalies
**Refractory anemia (RA)**	• Anemia<br>• No or rare blasts	• Erythroid dysplasia only<br>• <5% blasts, <15% ringed sideroblasts
**Refractory anemia with ring sideroblasts (RARS) ***	• Anemia<br>• No blasts	• Erythroid dysplasia only<br>• <5% blasts, >=15% ringed sideroblasts
**Refractory cytopenia with multilineage dysplasia (RCMD)**	• Bi- or pancytopenia<br>• No or rare blasts<br>• No Auer rods<br>• Monocytes <1.000/µl	• Dysplasia in >=10% of cells in >=2 myeloid cell lines<br>• No Auer rods<br>• <15% ringed sideroblasts<br>• <5% blasts
**Refractory cytopenia with multilineage dysplasia and ringed sideroblasts (RCMD-RS)**	• Bi- or pancytopenia<br>• No or rare blasts<br>• No Auer rods<br>• Monocytes <1.000/µl	• Dysplasia in >=10% of cells in >=2 myeloid cell lines<br>• No Auer rods<br>• >15% ringed sideroblasts<br>• <5% blasts
**Refractory anemia with excess blasts-I (RAEB-I)**	• Cytopenias<br>• <5% blasts<br>• No Auer rods<br>• Monocytes <1.000/µl	• Unilineage or multlineage dysplasia<br>• 5-9% blasts<br>• No Auer rods
**Refractory anemia with excess blasts-II (RAEB-II)**	• Cytopenias<br>• 5-19% blasts<br>• Auer rods +/-<br>• Monocytes <1.000/µl	• Unilineage or multlineage dysplasia<br>• 10-19% blasts<br>• Auer rods +/-
**MDS unclassified (MDS-U)**	• Cytopenias<br>• No or rare blasts<br>• No Auer rods	• Unilineage dysplasia in granulocytes or MKs<br>• No Auer rods<br>• <5% blasts
**5q- syndrome**	• Anemia<br>• <5% blasts<br>• PLT normal or ↑	• Normal to increased MKs with hypolobulated nuclei<br>• No Auer rods<br>• <5% blasts<br>• Isolated del(5q)
**CMML-1 ****	• Monocytes >1.000/µl<br>• Ph-neg./ Bcr-Abl neg.<br>• <20% myeloblasts + monoblasts + promonocytes<br>• <5% blasts	• Dysplastic changes in >=1 myeloid lineage<br>• <20% myeloblasts + monoblasts + promonocytes or<br>• persistent monocytosis >3 months with excluxion of all other causes of monocytosis<br>• <10% blasts
**CMML-2 ****	• Monocytes >1.000/µl<br>• Ph-neg./ Bcr-Abl neg.<br>• <20% myeloblasts + monoblasts + promonocytes<br>• 5-19% blasts	• Dysplastic changes in >=1 myeloid lineage<br>• <20% myeloblasts + monoblasts + promonocytes or<br>• persistent monocytosis >3 months with excluxion of all other causes of monocytosis<br>• 10-19% blasts<br>• Auer rods +/-
**CMML-1 with eosinophilia**	• Criteria for CMML-1<br>• Eosinophils >1,500/µl	
**CMML-2 with eosinophilia**	• Criteria for CMML-2<br>• Eosinophils >1,500/µl	

* Ringed sideroblasts are not strong indicators for discrimination between CMPDs and MDS.

** Patients with WBC <=12,000/µl are generally considered to have MDS, whereas patients with WBC >12,000/µl are considered to have a CMPD.

Table 2: Risk stratification according to the International Prognostic Scoring System (IPSS)

Risk Group	*Score*	*Median survival (years)*	*Time to AML transformation in 25% of patients (years)*
Low risk	0	5,7	9.4
Intermediate 1	0.5 – 1.0	3,5	3.3
Intermediate 2	1.5 – 2.0	1,2	1.1
High risk	≥2,5	0,4	0.2

Score Value	*0*	*0.5*	*1*	*1.5*	*2*
BM blasts	<5	5-10		11-20	21-30
Karyotype	good	intermediate	poor		
Cytopenias	0/1	2/3			

Karyotype: Good: normal,-Y,del(5q),del(20q)
Poor:complex (≥3 abnormalities) or chromosome 5 or 7 anomalies
Intermediate: other abnormalities

Cytopenias: Hemoglobin <100 g/l
Neutrophils <1,8 G/l
Platelets <100 G/l

This Hypomethylating Agent Patient Registry is set up to collect real-world experience in the management of patients with myeloid neoplasms, in particularly in patients with MDS, CMML or AML, treated with hypomethylating agents in Austria and potentially other participating countries. This registry will collect data in a retrospective as well as in a prospective manner at various sites. The aim is to gain valuable insights on both efficacy and toxicity of these drugs in a routine clinical setting in patients with various comorbidities.

2. STUDY DESIGN

This Registry is a retrospective and prospective, observational, multi-center, multi-national research initiative. Data will be collected from all sites willing to participate. It is expected, that the main data-bulk will be obtained from approximately 20 sites. An estimated 1000 - 1500 patients are expected to be included; these numbers may be revised over time as interest and demand dictates.

No pre-defined visits, medical tests, laboratory tests, procedures, or interventions are required. Physicians who have already treated patients with hypomethylating agents or are planning to initiate treatment with an hypomethylating agent can include patient data in this registry. To help maintain patient confidentiality, each patient will be assigned a unique patient identifying number upon enrollment; this number will accompany the patient's medical and other registry information throughout the lifetime of the registry.

Electronic Case Report Forms (eCRF) will be used for data collection.

The coordinating investigator will designate a person who will enter all available data into the Electronic Case Report Forms. Data evaluation, statistical analysis and interpretation will only be permitted by the coordinating investigator and the person(s) designated by him.

Additionally from some patients with myeloid neoplasms, blood or tissue samples will be stored for further analyses. These samples will also be obtained from patients, who are not or not yet treated with hypomethylating agents to comprise a control.

3. OBJECTIVES:

- Number of Cycles and Dosage of VIDAZA® and/or DACOGEN® therapy
- Response evaluation:
    - hematological response
    - cytogenetic response (if data is available)
    - quantification of reduced need for transfusions
    - median response duration
- Toxicites:
    - Grade
    - Duration
    - Consequences
        - Hospitalization, treatment, etc.
        - Dose modifications or treatment discontinuations of hypomethylating agent
- Severe adverse reactions
- Overall survival
- Prognostic factors for overall survival and response

4. INCLUSION AND EXCUSION CRITERIA

Physicians will select appropriate patients for enrollment. Appropriate patients are expected to:
Begin with or already have received treatment with a hypomethylating agent
Be willing to provide informed consent

Due to the non-interventional design of this program there are no specific in- or exclusion criteria. Hypomethylating agents are contraindicated in patients with a known hypersensitivity to azacitidine or mannitol. Hypomethylating agents are also contraindicated in patients with advanced malignant hepatic tumors. Women of childbearing potential should be advised to avoid becoming pregnant while receiving treatment with hypomethylating agents. Women treated with hypomethylating agents should not nurse. Men should be advised to not father a child while receiving treatment with hypomethylating agents.

5. ASSESSMENTS

Assessments before start of treatment with a hypomethylating agent

- Collect informed consent for treatment with hypomethylating agent
- Patient characteristics
- Medical history
- Blood count

- Blood chemistry

Assessments for each treatment cycle with an hypomethylating agent
- Date, dose and duration of hypomethylating-agent therapy
- Concomitant medications (if relevant for disease modification, prophylactic antibiotics/antifungals/virostatics)
- Response evaluation
- Adverse events
- Blood count
- Blood chemistry

End of therapy
- Patient status
- Blood count / blood chemistry
- Response evaluation

Assessments after end of therapy with hypomethylating agent
- Patient status (alive, dead, disease status, disease modifying treatments, best supportive care measures)

6. ADVERSE EVENTS:

All adverse events during the treatment period will be reported on the eCRF. An adverse event is any adverse change from the patient´s baseline (pretreatment) condition, including intercurrent illness, which occurs during the course of a clinical trial after treatment has started, whether considered related to treatment or not (see “definition” below). Note: *planned* hospitalizations and p*rogression of the disease under study* will not be documented as serious adverse events.

The intensity (severity) of this event will be graded according to NCI Common Toxicity Criteria for Adverse Events (CTCAE) grading system in the toxicity categories that have recommended gradings.

This study will utilize the Common Terminology Criteria for Adverse Events v3.0 (CTCAE) for toxicity and serious adverse event reporting. If necessary, this may be updated to the most current version.
A copy of the CTC Version 3.0 can be downloaded from the CTEP home page (http://ctep.cancer.gov/protocolDevelopment/electronic_applications/docs/ctcaev3.pdf).

Adverse events not listed in the NCI CTC grading system will be graded according to a WHO four-point system as outlined below.

WHO Four-Point Scale – Definition of AE Severity
- Mild or Grade 1: discomfort noted, but no disruption to normal daily activities.
- Moderate or Grade 2: discomfort sufficient to reduce or affect normal daily activities
- Severe or Grade 3: Inability to work or perform normal daily activities
- Life-Threatening or Grade 4: Substantial risk of dying at time of event
- Death or Grade 5: Results in death

DEFINITION OF ADVERSE EVENT

Any untoward medical occurrence in a patient or clinical investigation subject administered a pharmaceutical product and which does not necessarily have to have a causal relationship with the treatment.
An adverse event can therefore be any unfavorable and unintended sign (including an abnormal laboratory finding), symptom, or disease temporally associated with the use of a medicinal product, whether or not considered related to the medicinal product.
Pre-existing conditions which worsen during a study are to be reported as adverse events. They can become Serious Adverse Events if they fulfill one of the seriousness criteria described below.

DEFINITION OF SERIOUS ADVERSE EVENT

Any adverse event (AE) that regardless of causal relationship to one of the study drugs at any dose fulfills at least one of the following criteria is considered a Serious Adverse Event (SAE):

- is fatal (results in death) (note: death is an outcome, not an event)
- is life-threatening (note: the term "life-threatening" refers to an event in which the patient was at risk of death at the time of the event; it does not refer to an event which could hypothetically have caused death had it been more severe).
- requires patient hospitalization or prolongation of existing hospitalization (note: "hospitalization" refers to an unplanned, overnight hospitalization).
- results in persistent or significant disability/incapacity.
- is a congenital anomaly/birth defect.
- is medically significant or requires intervention to prevent one or other of the outcomes listed above.
- New cancer

Events not considered to be SAEs are hospitalizations which: were planned before entry into the clinical study, are for elective treatment of a condition unrelated to the studied indication or its treatment, occur on an emergency outpatient basis and do not result in admission (unless fulfilling other criteria above), are part of the normal treatment or monitoring of the studied indication and are not associated with any deterioration in condition.

ASSESSMENT OF CAUSALITY

The causality of AEs and/or SAEs (i.e. their relationship to study treatment) will be assessed by the investigator(s) and documented in the case report form. Causality can be one of three possibilities:

- "NO" (definitely not drug related)
- "YES" (remotely, possibly, probably or definitely drug-related)
- "UNKNOWN"

All adverse events judged by either the investigator or the sponsor as being definitely not "NON DRUG-RELATED", do not qualify as Adverse Drug Reactions (ADR).

SERIOUS ADVERSE DRUG REACTION (SADR) REPORTING

All SADR should be documented in the eCRF by the respective participating site, as soon as the investigator becomes aware of the SADR. This information will be sent to the sponsor automatically upon saving the entered data in the eCRF.

The sponsor will inform Regulatory Authorities and the Ethics Committee according to Austrian law.

7. STATISTICAL ANALYSES

The overall approach of this non interventional protocol is descriptive. All parameters will be summarized using standard summary statistics as appropriate (n, mean, standard deviation, median, minimum, maximum, 25th percentile, and 75th percentile). Summary statistics for categorical variables will include frequency counts and percentages. Cox-regression stratified on the various factors will be used for univariate analyses of risk-factors for OS. For multivariate analysis Cox-regression with stepwise selection will be used. Additional statistical methods may become necessary.

8. INFORMED CONSENT

Written informed consent is to be obtained from each patient, whose data is entered into this registry prospectively. For those patients, whose data is collected retrospectively, written informed consent is to be obtained from all patients that are still alive.

All patients, from whom blood or tissue samples are stored for further analysis, will have to sign an additional informed consent for accompanying scientific research.

Samples from patients, who are not or not yet treated with a hypomethylating agent, will also be stored and comprise a control. These patients will only sign the additional informed consent for accompanying scientific research.

Patienteninformation und Einwilligungserklärung zur Teilnahme an einem Datenregister

Datenregister über Hypomethylierende Substanzen bei Patienten mit myeloiden Neoplasien, inklusive Myelodysplastischen Syndrom (MDS), Chronische Myelomonozytäre Leukämie (CMML) und Akute Myeloische Leukämie (AML)

Sehr geehrte Patientin, sehr geehrter Patient!

Ihr Arzt hat bei Ihnen eine Erkrankung aus dem myelodysplastischen Formenkreis festgestellt. Sie haben bereits oder werden eine Therapie mit einer hypomethylierenden Substanz zur Behandlung Ihrer Erkrankung erhalten. Vidaza ist ein Medikament, das in der Europäischen Union für die Behandlung von bestimmten Subgruppen von Patienten mit MDS, CMML und AML zugelassen wurde. Dacogen ist ein Medikament das vor Kurzem in der Europäischen Union für die Behandlung von AML zugelassen wurde.

Wir laden Sie ein, an oben genanntem Datenregister teilzunehmen. Ihre Teilnahme erfolgt freiwillig. Die Ablehnung der Teilnahme an diesem Register hat keine nachteiligen Folgen für Ihre medizinische Betreuung. Auch können Sie die Einwilligung zur Teilnahme jederzeit widerrufen. In diesem Fall werden die bis dahin erhobenen anonymisierten Daten weiterhin für wissenschaftliche Zwecke verwendet, aber es werden keine weiteren neuen Daten gesammelt.
Wenn Sie an diesem Register teilnehmen, hat das keinen unmittelbaren Einfluss auf die Art Ihrer Behandlung. Sie werden im Rahmen der Routine behandelt, und es ergeben sich für Sie durch die Teilnahme an diesem Register keine Änderungen. Sie geben lediglich die Zustimmung, dass Ihre routinemäßig erhobenen Daten in eine Datenbank eingegeben werden und zu wissenschaftlichen Zwecken verwendet werden können. Nur die AGMT (Arbeitsgemeinschaft medikamentöse Tumortherapie) und von der AGMT beauftragte Personen, die Mitarbeiter der klinischen Abteilungen, an denen dieses Datenregister durchgeführt wird, sowie in- und ausländische Gesundheitsbehörden haben Zugang zu den vertraulichen Daten, in denen Sie namentlich genannt werden. Diese Personen unterliegen der Schweigepflicht.

Die Daten werden kodiert aufgezeichnet (es werden nur eine Patientennummer und Ihr Geburtsjahr weitergegeben); eine Rückverfolgung zu Ihren persönlichen Daten kann nur durch autorisierte Mitarbeiter in der Abteilung erfolgen.

Erhebung der Lebensqualität:

Anhand eines Fragebogens wird die gesundheitsbezogene Lebensqualität erfasst. Sie werden deshalb gebeten, vor, während und nach der Therapie (etwa alle 3 Monate im Rahmen der Routineuntersuchungen) Fragebögen zur Erfassung Ihrer Lebensqualität auszufüllen.

Zu diesem Register sowie zur Patienteninformation und Einwilligungserklärung wurde von der zuständigen Ethikkommission eine befürwortende Stellungnahme abgegeben.

Durch Ihre Teilnahme an diesem Datenregister entstehen für Sie keine zusätzlichen Kosten.

Für weitere Fragen im Zusammenhang mit diesem Register stehen Ihnen Ihr behandelnder Arzt und seine Mitarbeiter gerne zur Verfügung.

Einwilligungserklärung

Name des Patienten in Druckbuchstaben: …………………………………………………………

Geb.Datum: ………………………………

Ich erkläre mich bereit, an dem Datenregister:
„Hypomethylierende Substanzen bei Patienten mit myeloiden Neoplasien, inklusive Myelodysplastischen Syndrom (MDS), CMML und AML“
teilzunehmen.

Beim Umgang mit den Daten werden die Bestimmungen des Datenschutzgesetzes 2000 beachtet.

Eine Kopie dieser Patienteninformation und Einwilligungserklärung habe ich erhalten. Das Original verbleibt beim Prüfarzt.

………………………………………………………………
(Datum und Unterschrift des Patienten)

………………………………………………………………
(Datum, Name und Unterschrift des verantwortlichen Arztes)

(Der Patient erhält eine unterschriebene Kopie der Patienteninformation und Einwilligungserklärung, das Original verbleibt beim Prüfarzt.)

References

1. Devlin N, Parkin D, Jenssen B. Methods for Analysing and reporting EQ-5D Data. Economic evaluation compares the costs and consequences of alternative courses of action.: Springer; 2020.
2. EuroQol, editor. Measuring Self-Reported Population Health: An International Perspective Based on Eq-5d: Spring Med Publishing; 2004.
3. EuroQol. Choosing a value setAccessed 14.12.2020. (accessed.
4. Feng Y, Devlin N, Bateman A, Zamora B, Parkin D. The Distribution of the EQ-5D-5L Index in Patient Populations2016. (accessed.
5. Szende A, Schaefer C, Goss TF, et al. Valuation of transfusion-free living in MDS: results of health utility interviews with patients. *Health Qual Life Outcomes* 2009; **7**: 81.
6. Oliva EN, Finelli C, Santini V, et al. Quality of life and physicians' perception in myelodysplastic syndromes. *Am J Blood Res* 2012; **2**(2): 136-47.
7. Stauder R, Yu G, Koinig KA, et al. Health-related quality of life in lower-risk MDS patients compared with age- and sex-matched reference populations: a European LeukemiaNet study. *Leukemia* 2018; **32**(6): 1380-92.
8. de Swart L, Crouch S, Hoeks M, et al. Impact of red blood cell transfusion dose density on progression-free survival in patients with lower-risk myelodysplastic syndromes. *Haematologica* 2020; **105**(3): 632-9.
9. Uyl-de Groot CA, Lowenberg B, Vellenga E, Suciu S, Willemze R, Rutten FF. Cost-effectiveness and quality-of-life assessment of GM-CSF as an adjunct to intensive remission induction chemotherapy in elderly patients with acute myeloid leukemia. *Br J Haematol* 1998; **100**(4): 629-36.
10. Slovacek L, Slovackova B, Jebavy L, Macingova Z. Psychosocial, health and demographic characteristics of quality of life among patients with acute myeloid leukemia and malignant lymphoma who underwent autologous hematopoietic stem cell transplantation. *Sao Paulo Med J* 2007; **125**(6): 359-61.
11. Leunis A, Redekop WK, Uyl-de Groot CA, Lowenberg B. Impaired health-related quality of life in acute myeloid leukemia survivors: a single-center study. *Eur J Haematol* 2014; **93**(3): 198-206.
12. Kurosawa S, Yamaguchi T, Mori T, et al. Patient-reported quality of life after allogeneic hematopoietic cell transplantation or chemotherapy for acute leukemia. *Bone Marrow Transplant* 2015; **50**(9): 1241-9.
13. van Dongen-Leunis A, Redekop WK, Uyl-de Groot CA. Which Questionnaire Should Be Used to Measure Quality-of-Life Utilities in Patients with Acute Leukemia? An Evaluation of the Validity and Interpretability of the EQ-5D-5L and Preference-Based Questionnaires Derived from the EORTC QLQ-C30. *Value Health* 2016; **19**(6): 834-43.
14. Mamolo CM, Cappelleri JC, Hoang CJ, et al. A real-world, cross-sectional, community survey of symptoms and health-related quality of life of adults with acute myeloid leukemia. *Future Oncol* 2019; **15**(16): 1895-909.
15. Horvath Walsh LE, Rider A, Piercy J, et al. Real-World Impact of Physician and Patient Discordance on Health-Related Quality of Life in US Patients with Acute Myeloid Leukemia. *Oncol Ther* 2019; **7**(1): 67-81.
16. Yu H, Zeng X, Sui M, et al. A head-to-head comparison of measurement properties of the EQ-5D-3L and EQ-5D-5L in acute myeloid leukemia patients. *Qual Life Res* 2020.
17. Peipert JD, Yount SE, Efficace F, et al. Validation of the Functional Assessment of Cancer Therapy-Leukemia instrument in patients with acute myeloid leukemia who are not candidates for intensive therapy. *Cancer* 2020; **126**(15): 3542-51.
18. Pratz KW, Panayiotidis P, Recher C, et al. Venetoclax combinations delay the time to deterioration of HRQoL in unfit patients with acute myeloid leukemia. *Blood Cancer J* 2022; **12**(4): 71.
19. EuroQol. EQ-5D-3L User Guide, Basic information on how to use the EQ-5D-3L instrument, Version 6.0December 2018. (accessed.
20. EuroQol. EQ-5D-5L User Guide, Basic information on how to use the EQ-5D-5L instrument, Version 3.0Sept 2019. (accessed.
21. Gerlinger C, Bamber L, Leverkus F, et al. Comparing the EQ-5D-5L utility index based on value sets of different countries: impact on the interpretation of clinical study results. *BMC Res Notes* 2019; **12**(1): 18.
22. Zrubka Z, Beretzky Z, Hermann Z, et al. A comparison of European, Polish, Slovenian and British EQ-5D-3L value sets using a Hungarian sample of 18 chronic diseases. *Eur J Health Econ* 2019; **20**(Suppl 1): 119-32.
23. Greiner W, Claes C, Busschbach JJ, von der Schulenburg JM. Validating the EQ-5D with time trade off for the German population. *Eur J Health Econ* 2005; **6**(2): 124-30.
24. Janssen MF, Birnie E, Haagsma JA, Bonsel GJ. Comparing the standard EQ-5D three-level system with a five-level version. *Value Health* 2008; **11**(2): 275-84.

25. Pickard AS, Kohlmann T, Janssen MF, Bonsel G, Rosenbloom S, Cella D. Evaluating equivalency between response systems: application of the Rasch model to a 3-level and 5-level EQ-5D. *Med Care* 2007; **45**(9): 812-9.
26. Devlin NJ, Krabbe PF. The development of new research methods for the valuation of EQ-5D-5L. *Eur J Health Econ* 2013; **14 Suppl 1**: S1-3.
27. Oppe M, Devlin NJ, van Hout B, Krabbe PF, de Charro F. A program of methodological research to arrive at the new international EQ-5D-5L valuation protocol. *Value Health* 2014; **17**(4): 445-53.
28. NICE. Position statement on use of the EQ-5D-5L value set for England (updated October 2019)Accesed 22.12.2020. (accessed.
29. Ludwig K, Graf von der Schulenburg JM, Greiner W. German Value Set for the EQ-5D-5L. *Pharmacoeconomics* 2018; **36**(6): 663-74.
30. Huber MB, Felix J, Vogelmann M, Leidl R. Health-Related Quality of Life of the General German Population in 2015: Results from the EQ-5D-5L. *Int J Environ Res Public Health* 2017; **14**(4).
31. Konig HH, Bernert S, Angermeyer MC. [Health Status of the German population: results of a representative survey using the EuroQol questionnaire]. *Gesundheitswesen* 2005; **67**(3): 173-82.
32. Hinz A, Klaiberg A, Brahler E, Konig HH. [The Quality of Life Questionnaire EQ-5D: modelling and norm values for the general population]. *Psychother Psychosom Med Psychol* 2006; **56**(2): 42-8.
33. Hinz A, Kohlmann T, Stobel-Richter Y, Zenger M, Brahler E. The quality of life questionnaire EQ-5D-5L: psychometric properties and normative values for the general German population. *Qual Life Res* 2014; **23**(2): 443-7.
34. van Hout B, Janssen MF, Feng YS, et al. Interim scoring for the EQ-5D-5L: mapping the EQ-5D-5L to EQ-5D-3L value sets. *Value Health* 2012; **15**(5): 708-15.
35. Grochtdreis T, Dams J, Konig HH, Konnopka A. Health-related quality of life measured with the EQ-5D-5L: estimation of normative index values based on a representative German population sample and value set. *Eur J Health Econ* 2019; **20**(6): 933-44.
36. Pickard AS, Neary MP, Cella D. Estimation of minimally important differences in EQ-5D utility and VAS scores in cancer. *Health Qual Life Outcomes* 2007; **5**: 70.
37. Henry EB, Barry LE, Hobbins AP, McClure NS, O'Neill C. Estimation of an Instrument-Defined Minimally Important Difference in EQ-5D-5L Index Scores Based on Scoring Algorithms Derived Using the EQ-VT Version 2 Valuation Protocols. *Value Health* 2020; **23**(7): 936-44.
38. Cheson BD, Greenberg PL, Bennett JM, et al. Clinical application and proposal for modification of the International Working Group (IWG) response criteria in myelodysplasia. *Blood* 2006; **108**(2): 419-25.
39. Dohner H, Estey E, Grimwade D, et al. Diagnosis and management of AML in adults: 2017 ELN recommendations from an international expert panel. *Blood* 2017; **129**(4): 424-47.
